# Age and sex adjusted adiposity estimators predict all cause and pneumonia related mortality in hospitalized older patients with severe dysphagia receiving artificial nutrition

**DOI:** 10.3389/fnut.2026.1791495

**Published:** 2026-02-25

**Authors:** Zhining Liu, Haiwei Chen, Jing Yang, Yongping Lu, Jie Li, Zeru Chen, Ming Jin

**Affiliations:** 1 Department of Emergency Medicine, Guangyuan Central Hospital, Guangyuan, Sichuan, China; 2 Guangzhou Medical University, Guangzhou, Guangdong, China; 3 Guangyuan Central Hospital, Guangyuan, Sichuan, China

**Keywords:** CUN-BAE, Deurenberg, ECORE-BF, mortality, severe dysphagia

## Abstract

**Background:**

Older adults with severe dysphagia who require percutaneous endoscopic gastrostomy (PEG) feeding and/or total parenteral nutrition (TPN) have substantial mortality risk, yet practical tools for prognostic stratification are limited. Whether age- and sex-adjusted adiposity estimation formulas (CUN-BAE, ECORE-BF, and the Deurenberg formula) can improve risk prediction for all-cause and pneumonia-related mortality in this setting remains unclear.

**Methods:**

This study is a secondary analysis of a previously established single-center Japanese retrospective cohort of 247 patients aged ≥50 years with severe dysphagia receiving percutaneous endoscopic gastrostomy (PEG) and/or total parenteral nutrition (TPN). Associations of adiposity estimators with all-cause and pneumonia-related mortality were evaluated using Kaplan–Meier analysis, Cox regression, restricted cubic splines (RCS), time-dependent ROC analysis, and the C-index. Incremental predictive value beyond the baseline model was assessed using net reclassification improvement (NRI) and integrated discrimination improvement (IDI), with sensitivity analyses including multiple imputation, exclusion of deaths within 30 days, and additional adjustment for the Clinical Frailty Scale.

**Results:**

Across tertiles, Kaplan–Meier curves separated significantly for both all-cause and pneumonia-related mortality. In fully adjusted Cox models, the highest tertile was associated with higher all-cause mortality (HR 2.02–2.33) and markedly higher pneumonia-related mortality (HR 3.78–5.09) compared with the lowest tertile, with evidence of monotonic trends. Restricted cubic spline analyses supported largely linear dose–response relationships. Predictive discrimination improved over time; at 3 years, CUN-BAE and Deurenberg showed higher AUCs than ECORE-BF for both endpoints. Incremental analyses indicated added value for all-cause mortality with CUN-BAE and Deurenberg.

**Conclusion:**

Adiposity estimation formulas, particularly CUN-BAE and Deurenberg, provide clinically useful mortality risk stratification in severe dysphagia receiving PEG/TPN.

## 1 Introduction

Severe dysphagia in older adults represents a growing clinical and nutritional care burden (1). As populations age, swallowing impairment related to stroke, neurodegenerative conditions, and frailty is encountered with increasing frequency, with important consequences for patient-centered outcomes and downstream prognosis (2–4). In the United States, published estimates suggest that dysphagia affects roughly 0.3–0.6 million individuals each year (5). Clinically, advanced dysphagia commonly results in inadequate oral intake and is often accompanied by malnutrition, derangements in fluid and electrolyte balance, and recurrent aspiration events, including aspiration pneumonia—factors that collectively contribute to higher rates of rehospitalization and mortality (1, 6). When oral intake is insufficient, sustained artificial nutrition support becomes necessary, typically through PEG tube feeding or TPN (7–9). Despite the central role of these interventions, prognostic stratification at the point of initiating, maintaining, and monitoring long-term nutrition support remains challenging, and widely applicable, objective tools to distinguish patients with substantially different mortality risks are still lacking.

Tools routinely used to describe nutritional status in practice, such as body mass index and short-term weight change, may be particularly limited in very old patients with severe dysphagia (10, 11). Aging is accompanied by shifts in body compartments (notably loss of skeletal muscle), variable fluid status (e.g., edema or dehydration), and chronic low-grade inflammation, which can obscure meaningful differences in body composition and reduce the prognostic informativeness of simple height–weight metrics. Consequently, bedside-friendly indicators that incorporate age and sex to better approximate body fatness may offer a more suitable basis for risk stratification in this setting (12–14).

Accordingly, several anthropometry-based adiposity estimators incorporating age and sex have been proposed, including CUN-BAE, ECORE-BF, and the Deurenberg formula. These approaches use mathematical models to estimate adiposity and may capture inter-individual heterogeneity in body composition even when overall body size appears similar, potentially adding clinically relevant information beyond conventional measures (15–17). While associations between these estimators and cardiometabolic risk as well as all-cause mortality have been reported in community samples and selected chronic disease cohorts (18, 19), evidence in older patients with severe dysphagia who require PEG feeding and/or TPN remains sparse. Moreover, aspiration pneumonia constitutes a major and clinically actionable cause of death in this population (20), yet the relationships between adiposity estimators and pneumonia-specific mortality—and the relative prognostic performance of different formulas for all-cause vs. pneumonia-related outcomes—have not been rigorously evaluated.

Therefore, using a retrospective cohort of older patients with dysphagia receiving PEG and/or TPN, this study examined associations of three adiposity estimators with all-cause and pneumonia-related mortality, and compared their discriminative and predictive performance to inform practical, clinically relevant risk stratification.

## 2 Materials and methods

### 2.1 Data source and ethical approval

Patient-level data were obtained from a previously established single-center registry in Japan previously reported by Masaki and Shigenori (9) limited to records collected between January 2014 and January 2017. The dataset was made publicly available by the original authors as an open-access resource. The present study represents a secondary analysis of this publicly available dataset. The source publication is distributed under the Creative Commons Attribution license, permitting reuse with appropriate credit to the original authors and the source. The original study was approved by the Ethics Review Committee of Miyanomori Memorial Hospital and was conducted in accordance with the Declaration of Helsinki and relevant guidelines and regulations. As current analysis involved only de-identified public data, no additional ethical approval was required.

### 2.2 Study population

Eligibility criteria were patients aged ≥50 years with severe dysphagia who received nutrition support by percutaneous endoscopic gastrostomy (PEG) or total parenteral nutrition (TPN) during the study period. Patients were excluded if they had advanced malignancy, if PEG was performed for gastric decompression, or if PEG placement occurred before 2014. One additional case was excluded due to missing BMI-related nutritional measures (*n* = 1). The final analytic sample comprised 247 participants.

### 2.3 Data collection and variable definitions

#### 2.3.1 Baseline data and covariates

The initiation date of nutritional support was used as the baseline time point. Baseline information was compiled using a pre-specified scheme and covered demographic characteristics, measures reflecting nutritional status and the delivery of nutritional support, pre-existing comorbidities, and laboratory test results. Comorbidities were grouped according to diagnoses documented in the medical records. Laboratory parameters were restricted to measurements obtained within the 7 days preceding initiation; when more than one value was available for the same parameter within this window, the measurement closest to the baseline date was selected for analysis. Detailed definitions, units, and abbreviations are provided in Supplementary Table S1.

#### 2.3.2 Exposure: BMI-related nutritional indices

The exposures of interest were BMI-derived indices for nutritional/adiposity assessment, namely CUN-BAE (21), ECORE-BF (16), and the Deurenberg equation (22). These indices were calculated from baseline BMI, age, and sex. The corresponding formulas are shown in Table 1.

**Table 1:** BMI-related nutritional indices.

**Name of equation/ Anthropometric measurement**	**Equation/Units**
Body mass index (BMI)	Weight (kg)/[height (m)]^2^
University of Navarra Clinic-Body Fat Estimator (CUN-BAE) index	BF% = −44.988+(0.503 × age (yrs)) + (10.689 × sex^#^) + (3.172 × BMI) – (0.026 × BMI^2^) + (0.181 × BMI × sex) – (0.02 × BMI × age (yrs)) – (0.005 × BMI2 × sex) + (0.00021 × BMI^2^ × age (yrs))
Equation Córdoba for Estimation of Body Fat (ECORE-BF)	BF% = −97.102 + (0.123 × age (yrs)) + (11.900 × sex^#^) + (35.959 × (LnBMI))
Equation by Deurenberg et al. (22)	BF% = 1.2 × BMI + 0.23 × age (years) −10.8 × sex^*^ – 5.4

^*^Sex = 1 for males, 0 for females.

^#^Sex = 0 for male, 1 for female.

### 2.4 Clinical outcomes

The primary endpoint was all-cause mortality, defined as death from any cause. Survival time was calculated from the date of initiation of nutritional support to the date of death. The secondary endpoint was pneumonia-related mortality, defined as death attributable to pneumonia. Information on cause of death was obtained from hospital medical records and discharge summaries in the original registry. Pneumonia-related mortality was classified when pneumonia was documented by the treating physicians as the primary cause of death based on clinical, laboratory, and radiographic findings. In cases where multiple causes of death were recorded, pneumonia was considered the cause of death only if it was listed as the leading or underlying cause. Deaths with uncertain or insufficient information regarding pneumonia were not classified as pneumonia-related mortality.

### 2.5 Statistical analysis

Analyses were performed in R (v4.3.1). Categorical data are reported as *n* (%). Continuous variables are summarized as mean (SD) when approximately symmetric and as median (IQR) otherwise. For descriptive comparisons across outcome groups, ANOVA was used for continuous variables and chi-square tests for categorical variables; Fisher's exact test was substituted where sparse cells occurred.

Associations between BMI-related indices and time-to-death outcomes were evaluated with Cox regression, separately for all-cause mortality and pneumonia-related mortality, yielding hazard ratios (HRs) with 95% confidence intervals (CI). When adiposity estimators derived from age, sex, and body mass index (CUN-BAE, ECORE-BF, and the Deurenberg equation) were included as exposure variables, age, sex, and body mass index were not additionally adjusted for, because these variables are intrinsic components of the estimation formulas; additional adjustment would therefore constitute overadjustment and could introduce multicollinearity. Before fitting multivariable models, covariate collinearity was screened using generalized variance inflation factors (GVIFs) and the condition index, with no concerning collinearity observed (Supplementary Table S2). The proportional hazards assumption was assessed using Schoenfeld residual-based tests (Supplementary Table S3).

Survival patterns across exposure levels were visualized with Kaplan–Meier curves and compared by log-rank tests. Potential departures from linearity were explored using restricted cubic splines (RCS); likelihood ratio testing was used to evaluate the non-linear component (reported as *P* for non-linearity). RCS models were fitted using three knots placed at the 10th, 50th, and 90th percentiles of the corresponding adiposity estimator distributions, in accordance with commonly used recommendations.

Effect modification was examined through pre-specified subgroup analyses (e.g., age, sex, comorbidity strata) and by adding interaction terms; interaction *P* values followed recommended methodological practice (23, 24), and subgroup results are presented as forest plots. Predictive performance of CUN-BAE, ECORE-BF, and Deurenberg for 1-, 2-, and 3-year risks of all-cause and pneumonia-related death was compared using time-dependent ROC analysis. Added predictive value beyond a reference model was quantified using net reclassification improvement (NRI) and integrated discrimination improvement (IDI). Continuous NRI, without pre-specified risk categories, was applied to evaluate improvement in risk prediction for time-to-event outcomes.

Robustness checks included: repeating analyses after multiple imputation by chained equations (25) for missing covariates (Supplementary Tables S12, S13); repeating analyses after excluding deaths within 30 days of baseline (Supplementary Tables S14, S15); and additionally adjusting for the Clinical Frailty Scale (CFS; Supplementary Tables S16, S17). Two-sided *P* values were used, with *P* < 0.05 indicating statistical significance unless stated otherwise.

## 3 Results

### 3.1 Comparison of baseline characteristics between survivors and non-survivors

Baseline characteristics stratified by survival status are shown in Table 2. In total, 247 patients were included (mean age, 83.0 ± 9.3 years), and 151 (61.1%) were male. Relative to survivors, non-survivors were older and demonstrated a less favorable clinical profile. Regarding nutritional support modality, PEG was less frequently used among non-survivors, who were more often managed with TPN and also had a lower energy intake. The burden of comorbidities was higher in the non-survival group, with greater proportions of cardiovascular disease (CVD), severe dementia, aspiration pneumonia (Asp), ischemic heart disease (IHD), congestive heart failure (CHF), and chronic kidney disease (CKD). Laboratory data were consistent with poorer nutritional reserve and a more pronounced inflammatory state among non-survivors, including lower serum albumin (ALB), total lymphocyte count (TLC), total cholesterol (TC), and hemoglobin, together with higher C-reactive protein (CRP). The remaining variables were comparable between groups. Notably, BMI did not differ between groups; however, BMI-derived adiposity indices (CUN-BAE, ECORE-BF, and the Deurenberg formula) were higher in non-survivors.

**Table 2:** Baseline characteristics of patients with dysphagia according to all-cause mortality status.

**Variables**	**Total**	**Alive**	**Death**	***P*-value**
	**(*****n*** = **247)**	**(*****n*** = **114)**	**(*****n*** = **133)**	
CUN-BAE, Mean ± SD	27.4 ± 7.5	25.4 ± 6.8	29.1 ± 7.6	**< 0.001**
ECORE-BF, Mean ± SD	23.49 ± 8.98	21.85 ± 8.36	24.90 ± 9.29	**0.008**
Deurenberg, Mean ± SD	30.2 ± 7.0	28.2 ± 6.3	31.8 ± 7.2	**< 0.001**
**Demographic**				
Age (years), Mean ± SD	83.0 ± 9.3	80.4 ± 10.3	85.3 ± 7.7	**< 0.001**
Gender (Male), *n* (%)	151 (61.1)	86 (75.4)	65 (48.9)	**< 0.001**
**Nutritional parameters**				
BMI, Mean ± SD	19.2 ± 3.3	19.4 ± 3.4	19.0 ± 3.3	0.379
TPN, *n* (%)	67 (27.1)	10 (8.8)	57 (42.9)	**< 0.001**
PEG, *n* (%)	180 (72.9)	104 (91.2)	76 (57.1)	**< 0.001**
NT.CVC, *n* (%)	22 (8.9)	1 (0.9)	21 (15.8)	
Nutrient intake, Kcal/day	917.7 ± 187.5	963.9 ± 145.8	878.0 ± 209.5	**< 0.001**
**Comorbidities**				
CVD, *n* (%)	132 (53.4)	71 (62.3)	61 (45.9)	**0.010**
Severe dementia, *n* (%)	99 (40.1)	32 (28.1)	67 (50.4)	**< 0.001**
NMD, *n* (%)	14 (5.7)	7 (6.1)	7 (5.3)	0.766
Asp, *n* (%)	92 (37.2)	32 (28.1)	60 (45.1)	**0.006**
IHD, *n* (%)	44 (17.8)	12 (10.5)	32 (24.1)	**0.006**
CHF, *n* (%)	102 (41.3)	31 (27.2)	71 (53.4)	**< 0.001**
CPD, *n* (%)	18 (7.3)	6 (5.3)	12 (9)	0.257
CLD, *n* (%)	15 (6.1)	5 (4.4)	10 (7.5)	0.304
CKD, *n* (%)	52 (21.1)	9 (7.9)	43 (32.3)	**< 0.001**
**Lab biomarkers**				
ALB (g/dl), Mean ± SD	3.1 ± 0.6	3.4 ± 0.6	2.9 ± 0.6	**< 0.001**
TLC (10^9^/L), Mean ± SD	1.3 ± 0.7	1.5 ± 0.6	1.2 ± 0.8	**0.001**
TC (mg/dl), Mean ± SD	156.1 ± 40.2	167.2 ± 37.0	147.2 ± 40.6	**< 0.001**
Hemoglobin (g/dl), Mean ± SD	11.0 ± 2.0	11.8 ± 1.8	10.3 ± 1.9	**< 0.001**
CRP (mg/L), Median (IQR)	1.0 (0.3, 3.2)	0.6 (0.2, 1.6)	1.7 (0.5, 4.2)	**< 0.001**
**Outcomes**				
Oral intake recovery, *n* (%)	14 (5.7)	14 (12.3)	0 (0)	**< 0.001**
Discharge to home, *n* (%)	38 (15.4)	23 (20.2)	15 (11.3)	0.053
Severe sepsis, *n* (%)	29 (11.9)	3 (2.7)	26 (19.8)	**< 0.001**
Severe pneumonia, *n* (%)	68 (27.8)	10 (8.8)	58 (43.9)	**< 0.001**

CVD, cerebrovascular diseases; NMD, neuromuscular diseases; Asp, previous history of aspiration pneumonia; IHD, ischemic heart diseases; CHF, chronic heart failure; CPD, chronic pulmonary disease, CLD, chronic liver diseases; CKD, chronic kidney diseases; ALB, serum albumin; TLC, total lymphocyte count; TC, total cholesterol; CRP, C-reactive protein; NT.CVC, non-tunneled central venous catheters; PEG, percutaneous endoscopic gastrostomy; TPN, total parenteral nutrition; BMI, body mass index; CUN-BAE, Clínica Universidad de Navarra-Body Adiposity Estimator; ECORE-BF, Córdoba Equation for Estimation of Body Fat.

P-value less than 0.05 is expressed in bold.

### 3.2 Kaplan–Meier survival curves for all-cause and pneumonia-specific mortality across tertiles of BMI-related adiposity indices

**Figure 1:**
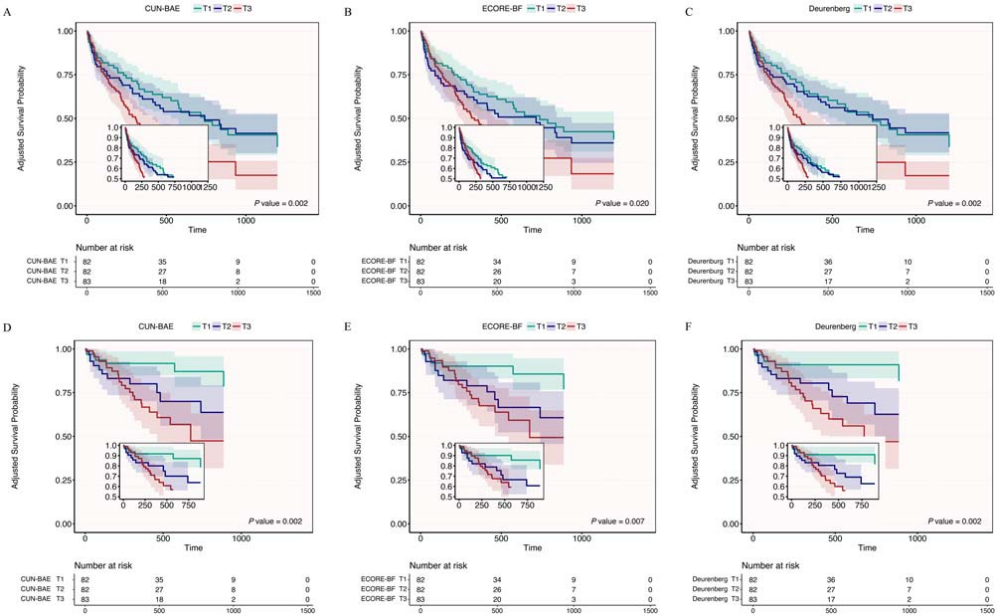
Adjusted survival curves derived from the fully adjusted Cox proportional hazards model. Adjusted survival curves for all-cause mortality **(A–C)** and pneumonia-related mortality **(D–F)** according to tertiles (T1–T3) of CUN-BAE **(A, D)**, ECORE-BF **(B, E)**, and the Deurenberg estimator **(C, F)**. Curves represent adjusted survival probabilities derived from the fully adjusted Cox model; shaded bands indicate 95% confidence intervals. Numbers at risk are shown below each panel; insets display an enlarged view of the early follow-up period. CUN-BAE, Clínica Universidad de Navarra–Body Adiposity Estimator; ECORE-BF, Equation Córdoba for Estimation of Body Fat; T1–T3, tertiles.

As shown in Figure 1, clear survival differences were observed across tertiles of the three BMI-derived adiposity indices. For all-cause mortality (Figures 1A–C), the curves separated in a graded manner, with survival progressively decreasing from the lowest to the highest tertile; between-group differences were significant (CUN-BAE: *P* = 0.002; ECORE-BF: *P* = 0.020; Deurenberg: *P* = 0.002). A concordant pattern was also seen for pneumonia-specific mortality (Figures 1D–F), where participants in higher tertiles consistently exhibited poorer survival (log-rank tests, all *P* < 0.01). Collectively, higher BMI-derived adiposity estimates were associated with unfavorable outcomes for both endpoints.

### 3.3 Association of BMI-related adiposity indices with all-cause mortality

Multivariable Cox regression results for all-cause mortality are summarized in Table 3. In the fully adjusted model, all three indices were independently related to mortality risk and demonstrated a dose-graded pattern. When analyzed by tertiles, the highest tertile (T3) showed an approximately twofold higher hazard compared with T1: CUN-BAE (HR of 2.33, 95% CI from 1.40 to 3.89), ECORE-BF (HR of 2.02, 95% CI from 1.22 to 3.32), and the Deurenberg index (HR of 2.30, 95% CI from 1.37 to 3.88), with all comparisons reaching statistical significance (*P* < 0.01). Trend tests further supported an increasing risk across tertiles (all *P* for trend ≤ 0.006). These findings indicate that BMI-derived adiposity indices provide consistent and independent prognostic information for all-cause mortality.

**Table 3:** Association between nutritional indicators related to BMI index and all-cause mortality across multivariable models.

**Variables**	**Total**	**Event**	**Model 1**		**Model 2**		**Model 3**	
			**HR (95% CI)**	***P*** **value**	**HR (95% CI)**	***P*** **value**	**HR (95% CI)**	***P*** **value**
**CUN-BAE**	247	133 (53.8)	1.05 (1.03–1.07)	< 0.001	1.05 (1.02–1.07)	< 0.001	1.06 (1.03–1.09)	< 0.001
T1	82	37 (45.1)	1.0 [Ref]		1.0 [Ref]		1.0 [Ref]	
T2	82	37 (45.1)	1.09 (0.69–1.72)	0.718	1.12 (0.71–1.77)	0.633	1.14 (0.69–1.88)	0.610
T3	83	59 (71.1)	2.18 (1.44–3.30)	< 0.001	2.15 (1.40–3.31)	< 0.001	2.33 (1.40–3.89)	0.001
*P-*trend	247	133 (53.8)	1.51 (1.22–1.87)	< 0.001	1.48 (1.19–1.85)	< 0.001	1.53 (1.18–1.99)	0.001
**ECORE-BF**	247	133 (53.8)	1.03 (1.01–1.05)	0.002	1.03 (1.01–1.05)	0.004	1.04 (1.01–1.06)	0.002
T1	82	40 (48.8)	1.0 [Ref]		1.0 [Ref]		1.0 [Ref]	
T2	82	36 (43.9)	1.00 (0.64–1.57)	0.990	1.06 (0.68–1.67)	0.796	1.44 (0.88–2.35)	0.150
T3	83	57 (68.7)	1.83 (1.22–2.74)	0.004	1.90 (1.25–2.89)	0.003	2.02 (1.22–3.32)	0.006
*P-*trend	247	133 (53.8)	1.37 (1.11–1.70)	0.003	1.39 (1.12–1.73)	0.003	1.42 (1.11–1.82)	0.006
**Deurenberg**	247	133 (53.8)	1.06 (1.03–1.08)	< 0.001	1.05 (1.03–1.08)	< 0.001	1.06 (1.03–1.09)	< 0.001
T1	82	35 (42.7)	1.0 [Ref]		1.0 [Ref]		1.0 [Ref]	
T2	82	38 (46.3)	1.24 (0.78–1.96)	0.363	1.16 (0.73–1.84)	0.527	1.07 (0.65–1.78)	0.780
T3	83	60 (72.3)	2.46 (1.61–3.75)	< 0.001	2.31 (1.49–3.58)	< 0.001	2.30 (1.37–3.88)	0.002
*P-*trend	247	133 (53.8)	1.60 (1.29–1.99)	< 0.001	1.55 (1.23–1.94)	< 0.001	1.53 (1.17–2.01)	0.002

Model 1: no covariates were adjusted.

Model 2: CVD, Severe dementia, NMD, IHD were adjusted.

Model 3: CVD, Severe dementia, NMD, IHD, Asp, CHF, CLD, CPD, CKD, NT.CVC, PEG, oral intake recovery, CRP, ALB, TLC, TC, Nutrient intake, Hemoglobin.

CVD, cerebrovascular diseases; NMD, neuromuscular diseases; Asp, previous history of aspiration pneumonia; IHD, ischemic heart diseases; CHF, chronic heart failure; CPD, chronic pulmonary disease, CLD, chronic liver diseases; CKD, chronic kidney diseases; ALB, serum albumin; TLC, total lymphocyte count; TC, total cholesterol; CRP, C-reactive protein; NT.CVC, non-tunneled central venous catheters; PEG, percutaneous endoscopic gastrostomy; TPN, total parenteral nutrition; BMI, body mass index; CUN-BAE, Clínica Universidad de Navarra-Body Adiposity Estimator; ECORE-BF, Córdoba Equation for Estimation of Body Fat; HR, hazard ratio; CI, confidence interval; Ref, reference.

### 3.4 Association of BMI-related adiposity indices with pneumonia-specific mortality

Cox regression results for pneumonia-related mortality are presented in Table 4. Relative to all-cause mortality, stronger associations were evident for pneumonia-related death. In the fully adjusted model, the hazard in T3 was markedly elevated compared with T1: CUN-BAE (HR of 4.81, 95% CI from 1.86 to 12.47), ECORE-BF (HR of 3.78, 95% CI from 1.48 to 9.68), and the Deurenberg index (HR of 5.09, 95% CI from 1.82 to 14.20); all were statistically significant (*P* < 0.01). A monotonic increase in risk was also supported by trend analyses (all *P* for trend ≤ 0.005). Overall, these BMI-derived indices effectively stratified the risk of pneumonia-related mortality, with a more pronounced gradient than that observed for all-cause death.

**Table 4:** Association between nutritional indicators related to BMI index and pneumonia-cause mortality across multivariable models.

**Variables**	**Total**	**Event**	**Model 1**		**Model 2**		**Model 3**	
			**HR (95% CI)**	***P value***	**HR (95% CI)**	***P value***	**HR (95% CI)**	***P value***
**CUN-BAE**	247	43 (17.4)	1.08 (1.04–1.12)	< 0.001	1.09 (1.04–1.13)	< 0.001	1.10 (1.05–1.16)	< 0.001
T1	82	8 (9.8)	1.0 [Ref]		1.0 [Ref]		1.0 [Ref]	
T2	82	12 (14.6)	1.66 (0.68–4.05)	0.269	1.82 (0.74–4.49)	0.195	3.15 (1.17–8.51)	0.024
T3	83	23 (27.7)	3.84 (1.71–8.62)	0.001	4.63 (2.02–10.63)	< 0.001	4.81 (1.86–12.47)	0.001
*P-*trend	247	43 (17.4)	2.02 (1.35–3.01)	0.001	2.21 (1.46–3.34)	< 0.001	2.11 (1.35–3.31)	0.001
**ECORE-BF**	247	43 (17.4)	1.05 (1.02–1.09)	0.002	1.06 (1.02–1.1)	0.001	1.07 (1.03–1.12)	0.001
T1	82	9 (11)	1.0 [Ref]		1.0 [Ref]		1.0 [Ref]	
T2	82	13 (15.9)	1.62 (0.69–3.79)	0.267	1.83 (0.78–4.33)	0.167	3.02 (1.2–7.63)	0.019
T3	83	21 (25.3)	2.94 (1.34–6.45)	0.007	3.62 (1.62–8.08)	0.002	3.78 (1.48–9.68)	0.006
*P-*trend	247	43 (17.4)	1.73 (1.18–2.54)	0.005	1.91 (1.29–2.84)	0.001	1.89 (1.21–2.95)	0.005
**Deurenberg**	247	43 (17.4)	1.08 (1.04–1.13)	< 0.001	1.08 (1.04–1.13)	< 0.001	1.10 (1.04–1.16)	0.001
T1	82	7 (8.5)	1.0 [Ref]		1.0 [Ref]		1.0 [Ref]	
T2	82	13 (15.9)	2.14 (0.85–5.37)	0.105	2.06 (0.81–5.19)	0.127	3.19 (1.11–9.16)	0.031
T3	83	23 (27.7)	4.60 (1.96–10.79)	< 0.001	5.12 (2.14–12.25)	< 0.001	5.09 (1.82–14.2)	0.002
*P-*trend	247	43 (17.4)	2.15 (1.43–3.21)	< 0.001	2.31 (1.51–3.52)	< 0.001	2.14 (1.33–3.43)	0.002

Model 1: no covariates were adjusted.

Model 2: CVD, Severe dementia, NMD, IHD were adjusted.

Model 3: CVD, Severe dementia, NMD, IHD, Asp, CHF, CLD, CPD, CKD, NT.CVC, PEG, oral intake recovery, CRP, ALB, TLC, TC, Nutrient intake, Hemoglobin.

CVD, cerebrovascular diseases; NMD, neuromuscular diseases; Asp, previous history of aspiration pneumonia; IHD, ischemic heart diseases; CHF, chronic heart failure; CPD, chronic pulmonary disease, CLD, chronic liver diseases; CKD, chronic kidney diseases; ALB, serum albumin; TLC, total lymphocyte count; TC, total cholesterol; CRP, C-reactive Protein; NT.CVC, Non-tunneled Central Venous Catheters; PEG, percutaneous endoscopic gastrostomy; TPN, total parenteral nutrition; BMI, body mass index; CUN-BAE, Clínica Universidad de Navarra-Body Adiposity Estimator; ECORE-BF, Córdoba Equation for Estimation of Body Fat; HR, hazard ratio; CI, confidence interval; Ref, reference.

### 3.5 Dose–response relationship between BMI-related adiposity indices and mortality

**Figure 2:**
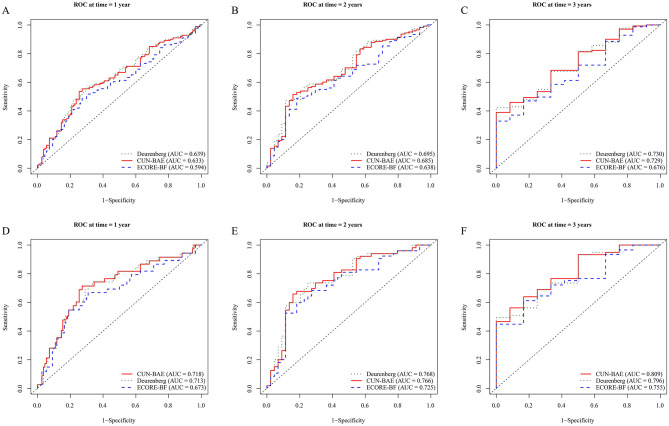
Restricted cubic spline analyses of adiposity indices and mortality risk. Restricted cubic spline (RCS) curves depicting the dose–response association between each adiposity index and all-cause mortality **(A–C)** or pneumonia-related mortality **(D–F)** in the fully adjusted Cox model. The solid line represents the estimated hazard ratio (HR) and the shaded area denotes the 95% confidence interval. The reference value (HR = 1.0) is indicated by the vertical dashed line; the horizontal dashed line denotes HR = 1.0. Histograms illustrate the distribution of the corresponding index. *P* values for the overall association and for non-linearity are reported within each panel. Restricted cubic spline models were fitted using three knots placed at the 10th, 50th, and 90th percentiles of each adiposity index. HR, hazard ratio; CI, confidence interval.

RCS analyses are shown in Figure 2. For all-cause mortality (Figures 2A–C), each index was significantly associated with mortality risk (overall association: CUN-BAE, *P* = 0.001; ECORE-BF, *P* = 0.021; Deurenberg, *P* = 0.002), and there was no evidence supporting non-linearity (all *P* for non-linearity > 0.05), suggesting an approximately linear increase in risk with higher index values.

Similar dose–response patterns were observed for pneumonia-specific mortality (Figures 2D–F), with significant overall associations (all *P* for overall < 0.01) and a more conspicuous risk elevation toward the upper range of the indices. Taken together, the RCS findings corroborate the Cox regression results and support a continuous, largely linear positive relationship between BMI-derived adiposity indices and mortality risk.

### 3.6 Predictive discrimination of BMI-derived adiposity indices for all-cause and pneumonia-related mortality

**Figure 3:**
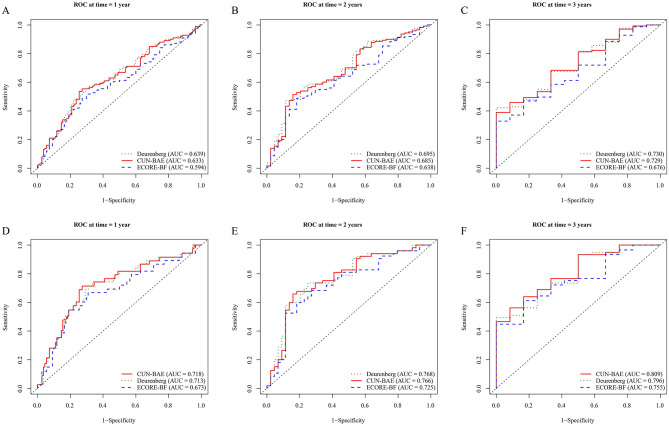
Time-dependent ROC curves for prediction of mortality at 1, 2, and 3 years. Time-dependent receiver operating characteristic (ROC) curves comparing the discrimination of CUN-BAE, ECORE-BF, and the Deurenberg estimator for predicting all-cause mortality at 1 year **(A)**, 2 years **(B)**, and 3 years **(C)**, and pneumonia-related mortality at 1 year **(D)**, 2 years **(E)**, and 3 years **(F)**. The area under the curve (AUC) for each index is displayed in the corresponding panel. ROC, receiver operating characteristic; AUC, area under the curve.

Time-dependent receiver operating characteristic (ROC) analyses were used to quantify the discriminatory performance of CUN-BAE, ECORE-BF, and the Deurenberg index at 1, 2, and 3 years (Figure 3; Supplementary Tables S6, S7). For all three indices, area under the ROC curve (AUC) values increased as the prediction horizon lengthened. With respect to all-cause mortality, the Deurenberg index and CUN-BAE consistently produced higher AUCs than ECORE-BF, with very similar estimates between the two leading indices (3-year AUC: 0.730 and 0.728 vs. 0.676). For pneumonia-related mortality, AUCs were generally higher than those observed for all-cause mortality; at 3 years, CUN-BAE and the Deurenberg index reached AUCs of 0.809 and 0.796, respectively, exceeding that of ECORE-BF (0.755; Supplementary Tables S6, S7).

Discrimination over the 1–3-year window was further evaluated using the concordance index (C-index; Supplementary Tables S8, S9). C-indices ranged from 0.57 to 0.61 across the three measures, with the Deurenberg index and CUN-BAE remaining slightly higher than ECORE-BF at each time point (e.g., at 2 years: 0.609 and 0.605 vs. 0.569), in agreement with the ranking suggested by the ROC analysis. Overall, all three BMI-derived indices demonstrated measurable discriminatory ability, with the Deurenberg index and CUN-BAE showing comparatively better performance, particularly for pneumonia-related mortality.

### 3.7 Incremental predictive value and clinical utility of BMI-related adiposity indices

Using the baseline risk model as the reference, incremental prognostic utility of CUN-BAE, ECORE-BF, and the Deurenberg index was quantified with NRI and IDI (Supplementary Tables S10, S11). For all-cause mortality, adding CUN-BAE yielded a statistically significant gain in both reclassification and discrimination (NRI = 0.468, *P* < 0.05; IDI = 0.028, *P* = 0.04). The Deurenberg index also improved reclassification (NRI = 0.475, *P* < 0.05) with a borderline increase in discrimination (IDI = 0.023, *P* = 0.06), whereas ECORE-BF did not materially enhance model performance (all *P* > 0.05). For pneumonia-related mortality, none of the three indices produced significant improvements in NRI or IDI. Overall, CUN-BAE and the Deurenberg index provided additional prognostic information for all-cause mortality beyond the baseline model, while their incremental value for pneumonia-related mortality appeared limited.

### 3.8 Sensitivity analyses

A series of sensitivity analyses was conducted to assess the robustness of the primary results and the stability of model specifications (Supplementary Figures S1–S6; Supplementary Tables S12–S17). In subgroup analyses, positive associations between the three BMI-derived adiposity indices and both all-cause and pneumonia-related mortality were broadly consistent across strata defined by age, sex, and major comorbidities, with no evidence of effect modification (all *P* for interaction >0.05). After addressing missingness via multiple imputation, effect estimates closely aligned with those of the primary analysis: under the fully adjusted model, the highest tertile was associated with increased all-cause mortality risk (CUN-BAE HR = 2.35; ECORE-BF HR = 1.96; Deurenberg HR = 2.31) and showed stronger associations with pneumonia-related mortality (HR = 4.18, 3.28, and 4.29, respectively; Supplementary Tables S12, S13). Exclusion of deaths occurring within 30 days did not change the direction of associations and slightly strengthened effect sizes, particularly for pneumonia-related death (T3 vs. T1: CUN-BAE HR = 6.89; ECORE-BF HR = 6.26; Deurenberg HR = 9.30; Supplementary Tables S14, S15). Additional adjustment for CFS did not materially alter the findings, and all three indices remained significantly associated with mortality outcomes (Supplementary Tables S16, S17). Collectively, these analyses indicate that the observed relationships between BMI-derived adiposity indices and both all-cause and pneumonia-related mortality are internally consistent and robust across analytic strategies.

## 4 Discussion

This study is the first to comprehensively examine the prognostic value of three body fat estimation equations based on body mass index, age, and sex—namely CUN-BAE, ECORE-BF, and the Deurenberg formula—in elderly patients with severe dysphagia receiving PEG or TPN. The findings indicate that higher estimated body fat levels are independently and approximately linearly associated with an increased risk of both all-cause mortality and pneumonia-related mortality. Among the evaluated models, the CUN-BAE and Deurenberg equations demonstrated superior predictive performance. Specifically, these indices provided significant incremental prognostic value beyond conventional predictors for all-cause mortality, as supported by continuous NRI and IDI analyses. For pneumonia-related mortality, although CUN-BAE and the Deurenberg index showed relatively strong discriminative ability based on time-dependent ROC and C-index analyses, no statistically significant incremental improvement was observed in NRI or IDI.

Nutritional support modality (PEG vs TPN) differed substantially between survivors and non-survivors in this cohort and is recognized as an important prognostic factor in clinical practice. In the present study, all patients received artificial nutritional support, and PEG and TPN were mutually exclusive, with their proportions summing to 100% within each outcome group. Accordingly, PEG use was included as a covariate in the multivariable Cox regression models, with non-PEG (TPN) as the reference category, to account for differences in nutritional support modality. This approach allows evaluation of the prognostic value of BMI-derived adiposity indices while accounting for the strong baseline clinical information conveyed by nutritional support choice.

Previous studies have demonstrated that BMI-derived body fat estimation formulas are useful for assessing metabolic risk and predicting all-cause mortality in community-dwelling populations and cohorts with cardiometabolic diseases (15, 26–28). However, their prognostic relevance in frail older patients with multimorbidity who depend on artificial nutritional support has remained largely unexplored. By extending the application of these tools to this particularly vulnerable population, our study identifies a key observation that differs from conventional understanding. Although a low BMI is generally regarded as a marker of malnutrition and adverse prognosis in older adults (29), baseline BMI did not differ significantly between survivors and non-survivors in our cohort, whereas all three estimated body fat indices were markedly higher among patients who died.

These findings suggest that, in the context of age-related alterations in body composition—characterized by progressive muscle loss and a relative increase in fat mass (30)—BMI alone is insufficient to capture the biological heterogeneity relevant to prognosis. In contrast, body fat estimation models adjusted for age and sex appear to more sensitively detect maladaptive adiposity signals associated with unfavorable outcomes. This observation offers a novel perspective for interpreting the frequently reported “obesity paradox” in critically ill older patients. While a modestly elevated BMI has traditionally been linked to improved survival (31, 32), such associations may obscure the underlying risk conferred by deteriorating body composition quality, particularly an excessive relative fat burden. Given that BMI cannot distinguish between lean and fat mass contributions, nor accurately reflect inter-individual differences across age and sex strata (33, 34), reliance on BMI alone may be misleading. Our results therefore indicate that higher adjusted body fat estimates independently predict poorer clinical outcomes, underscoring the importance of evaluating body composition quality rather than depending solely on anthropometric indices in this patient population.

The observed linear and independent associations between estimated body fat and both all-cause and pneumonia-related mortality in older patients with severe dysphagia likely reflect the convergence of multiple pathophysiological pathways, including mechanical respiratory impairment, systemic inflammatory–metabolic dysregulation, and the self-reinforcing interactions between these processes. Dysphagia directly increases the risk of aspiration, while excess body fat—particularly visceral adiposity—exacerbates thoracic mechanical load, restricts diaphragmatic excursion, and reduces pulmonary compliance, thereby compromising effective cough clearance and progressively increasing susceptibility to lower respiratory tract infections (35, 36).

Concurrently, adipose tissue expansion is accompanied by increased secretion of pro-inflammatory mediators, such as tumor necrosis factor-α and interleukin-6, which promote chronic low-grade inflammation and metabolic disturbances. These processes exert dose-dependent immunosuppressive effects, leading to a global impairment of host defense mechanisms (37, 38). Importantly, the coexistence of dysphagia and elevated body fat accelerates the development of sarcopenic obesity. On the one hand, increased adiposity and swallowing dysfunction jointly aggravate feeding difficulties and undernutrition, hastening skeletal muscle depletion. On the other hand, obesity-associated inflammation and insulin resistance further drive muscle wasting and functional decline. Together, these factors establish a vicious cycle characterized by physical frailty, immunosenescence, and progressive multi-organ dysfunction (39–41). As body fat accumulation and swallowing impairment advance in parallel, inflammatory burden, metabolic instability, and frailty severity intensify in a synergistic manner, resulting in a continuous and approximately linear escalation in vulnerability to pneumonia, infection severity, organ failure, and ultimately mortality risk (42, 43).

From a translational perspective, these findings carry clear clinical relevance. Both the CUN-BAE and Deurenberg equations rely solely on routinely available demographic and anthropometric variables, allowing for rapid calculation and straightforward integration into electronic medical record systems as automated bedside risk screening tools. Their application may inform multiple stages of clinical decision-making. Prior to the initiation of long-term artificial nutritional support, these indices can facilitate risk stratification and early identification of high-risk individuals, thereby supporting intensified surveillance, tailored nutritional strategies, and timely rehabilitative interventions. In addition, objective and quantifiable risk estimates may aid evidence-based prognostic communication and promote shared decision-making between clinicians, patients, and caregivers.

Several strengths of this study merit consideration. We focused on a particularly high-risk population—older adults with severe dysphagia requiring prolonged artificial nutritional support—in whom conventional nutritional assessment tools often perform suboptimally and clinical outcomes are poor, thereby addressing an important unmet clinical need. Methodologically, the use of multivariable Cox regression, restricted cubic spline modeling, time-dependent receiver operating characteristic analyses, and multiple sensitivity analyses enhances internal validity and strengthens the robustness of our conclusions.

Nevertheless, certain limitations should be acknowledged. The single-center, retrospective observational design introduces the possibility of selection bias. Moreover, as the cohort was derived from a single institution in Japan, the external generalizability of the findings warrants confirmation across diverse populations and healthcare settings. Finally, given the observational nature of the study, causal relationships between estimated body fat and adverse outcomes cannot be definitively established.

Future research should prioritize external validation in large, multicenter prospective cohorts and explore whether combining optimal body fat indices with other nutritional or functional markers can yield more refined prognostic models. In parallel, mechanistic investigations are needed to elucidate the biological pathways underlying elevated estimated body fat in this population. Ultimately, interventional trials will be required to determine whether comprehensive management strategies guided by body fat–based risk stratification can meaningfully improve clinical outcomes in older patients with severe dysphagia.

## 5 Conclusion

Across all three estimators, higher values were associated with greater risks of all-cause and pneumonia-related mortality, with largely linear relationships. Discriminative performance was generally better for pneumonia-related mortality than for all-cause mortality; however, incremental predictive improvement beyond the baseline model, as assessed by NRI and IDI, was observed only for all-cause mortality. CUN-BAE and the Deurenberg index showed more favorable overall performance metrics than ECORE-BF.

## Data Availability

The original contributions presented in the study are included in the article/Supplementary material, further inquiries can be directed to the corresponding authors.
