# Supplementary material for: Age and sex adjusted adiposity estimators predict all cause and pneumonia related mortality in hospitalized older patients with severe dysphagia receiving artificial nutrition
Source: Front Nutr. 2026 Feb 25;13:1791495. doi: 10.3389/fnut.2026.1791495 (PMC12975582; doi:10.3389/fnut.2026.1791495)
Supplement: Supplementary file 1 [file Table_1.docx]

Supplementary Material

**Table S1** The definition of the covariates

**Table S2** Assessment of multicollinearity across covariates.

**Table S3** Schoenfeld residual–based tests of the proportional hazards assumption for the fully adjusted Cox model (overall follow-up).

**Table S4** Baseline characteristics of patients with dysphagia according to pneumonia-related mortality status.

**Table S5** Association between nutritional indicators related to BMI index and sepsis-cause mortality across multivariable models.

**Table S6** The confidence Intervals and Area Under the Curve (AUC) Values for all-cause mortality.

**Table S7** The confidence Intervals and Area Under the Curve (AUC) Values for pneumonia-cause mortality.

**Table S8** C-Index values of nutritional indicators related to BMI for all-cause mortality.

**Table S9** C-Index values of nutritional indicators related to BMI for pneumonia-cause mortality.

**Table S10** Incremental Value of nutritional indicators related to BMI in all-cause mortality Prediction

**Table S11** Incremental Value of nutritional indicators related to BMI in pneumonia-cause mortality Prediction

**Table S12** Association Between nutritional indicators related to BMI and all-cause mortality Across Multivariable Models after MICE.

**Table S13** Association Between nutritional indicators related to BMI and pneumonia-cause mortality Across Multivariable Models after MICE.

**Table S14** Associations nutritional indicators related to BMI and all-cause mortality in patients with survival of more than 30 days.

**Table S15** Associations nutritional indicators related to BMI and pneumonia-cause in patients with survival of more than 30 days.

**Table S16** Association Between nutritional indicators related to BMI and all-cause mortality Across Additional adjusted for CFS in Multivariable Models.

**Table S17** Association Between nutritional indicators related to BMI and pneumonia-cause mortality Across Additional adjusted for CFS in Multivariable Models

**Fig. S1** C-index evaluation for nutritional indicators related to BMI in forecasting all-cause mortality.

**Fig. S2** Subgroup analysis of the relationship between CUN-BAE and all-cause mortality.

**Fig. S3** Subgroup analysis of the relationship between ECORE-BF and all-cause mortality.

**Fig. S4** Subgroup analysis of the relationship between Deurenberg and all-cause mortality.

**Fig. S5** Subgroup analysis of the relationship between CUN-BAE and pneumonia-cause mortality.

**Fig. S6** Subgroup analysis of the relationship between ECORE-BF and pneumonia-cause mortality.

**Fig. S7** Subgroup analysis of the relationship between Deurenberg and pneumonia-cause mortality.

**Table S1** The definition of the covariates

| **Variable** | **Definition / Measurement** | **Unit** | **Type** | **Notes** |
| --- | --- | --- | --- | --- |
| Age | Age at initiation of nutritional support | years | Continuous | Baseline variable |
| Sex | Male / Female as recorded in chart | — | Categorical | — |
| CFS (Clinical Frailty Scale) | Clinical frailty evaluated by attending physicians | score | Ordinal | 1–9 scale |
| BMI (Body Mass Index) | Body weight (kg) / height² (m²) | kg/m² | Continuous | — |
| Nutrition support type (NS) | PEG feeding or TPN | — | Binary | Exposure variable |
| Nutritional intake | Daily prescribed/actual energy or protein intake recorded at baseline | kcal/day or g/day | Continuous | If available |
| CVD | Presence of cardiovascular disease (ICD codes) | — | Binary | — |
| NMD | Diagnosed neuromuscular disorders | — | Binary | — |
| IHD | Clinical diagnosis of ischemic heart disease | — | Binary | — |
| CHF | History or ongoing treatment of chronic heart failure | — | Binary | — |
| CPD | Chronic pulmonary disease (e.g., COPD) | — | Binary | — |
| CLD | Chronic liver disease | — | Binary | — |
| CKD | Chronic kidney disease | — | Binary | — |
| ALB | Serum albumin within 7 days before PEG/TPN initiation | g/dL | Continuous | Baseline nutritional marker |
| TLC | Peripheral total lymphocyte count | ×10⁹/L | Continuous | Immune-nutrition marker |
| CRP | Serum C-reactive protein | mg/L | Continuous | Inflammatory marker |
| TC | Serum total cholesterol | mg/dL | Continuous | Metabolic indicator |
| Hb | Hemoglobin concentration | g/dL | Continuous | — |
| CUN-BAE | Calculated from BMI, age, and sex | % | Continuous | Body-fat estimation |
| ECORE-BF | Calculated from BMI, age, and sex | % | Continuous | Body-fat estimation |
| Deurenberg | Calculated from BMI, age, and sex | % | Continuous | Body-fat estimation |

**Table S2** Assessment of multicollinearity across covariates.

| Term | GVIF | DF | GVIF^(1/(2*Df)) | Colinearity (0=No, 1=Yes) |
| --- | --- | --- | --- | --- |
| PEG | 2.078 | 1 | 1.442 | 0 |
| NT.CVC | 1.813 | 1 | 1.346 | 0 |
| Nutrient intake, Kcal/day | 1.782 | 1 | 1.335 | 0 |
| CVD | 1.938 | 1 | 1.392 | 0 |
| Severe dementia | 2.067 | 1 | 1.438 | 0 |
| NMD | 1.482 | 1 | 1.217 | 0 |
| Asp | 1.585 | 1 | 1.259 | 0 |
| IHD | 1.577 | 1 | 1.256 | 0 |
| CHF | 1.564 | 1 | 1.251 | 0 |
| CPD | 1.164 | 1 | 1.079 | 0 |
| CLD | 1.287 | 1 | 1.134 | 0 |
| CKD | 1.831 | 1 | 1.353 | 0 |
| ALB | 1.562 | 1 | 1.250 | 0 |
| TLC | 1.235 | 1 | 1.111 | 0 |
| TC | 1.623 | 1 | 1.274 | 0 |
| Hemoglobin | 1.813 | 1 | 1.346 | 0 |
| CRP | 1.345 | 1 | 1.160 | 0 |
| Oral intake recovery | 1.000 | 1 | 1.000 | 0 |

Abbreviation: CVD, cerebrovascular diseases; NMD, neuromuscular diseases; Asp, previous history of aspiration pneumonia; IHD, ischemic heart diseases; CHF, Chronic Heart Failure; CPD, Chronic Pulmonary Disease, CLD, Chronic Liver Diseases; CKD, Chronic Kidney Diseases; ALB, serum albumin; TLC, Total Lymphocyte Count; TC, Total Cholesterol; CRP, C-reactive Protein; NT.CVC, Non-tunneled Central Venous Catheters; PEG, Percutaneous Endoscopic Gastrostomy.

**Table S3** Schoenfeld residual–based tests of the proportional hazards assumption for the fully adjusted Cox model (overall follow-up).

| **Variable** | **chisq** | **df** | **p.value** |
| --- | --- | --- | --- |
| **PEG** | 5.058 | 1 | 0.025 |
| **NT.CVC** | 3.950 | 1 | 0.047 |
| **CVD** | 1.393 | 1 | 0.238 |
| **Severe dementia** | 1.723 | 1 | 0.189 |
| **NMD** | 0.849 | 1 | 0.357 |
| **Asp** | 1.421 | 1 | 0.233 |
| **IHD** | 1.557 | 1 | 0.212 |
| **CHF** | 2.045 | 1 | 0.153 |
| **CPD** | 1.976 | 1 | 0.160 |
| **CLD** | 1.067 | 1 | 0.302 |
| **CKD** | 2.579 | 1 | 0.108 |
| **ALB** | 1.100 | 1 | 0.294 |
| **TLC** | 0.050 | 1 | 0.822 |
| **TC** | 0.001 | 1 | 0.974 |
| **Hemoglobin** | 0.022 | 1 | 0.881 |
| **CRP** | 0.352 | 1 | 0.553 |
| **Nutrient intake, Kcal/day** | 1.254 | 1 | 0.263 |
| **Oral intake recovery** | 1.161 | 1 | 0.281 |
| **Deurenberg** | 0.686 | 1 | 0.408 |
| **ECORE-BF** | 0.393 | 1 | 0.531 |
| **CUN-BAE** | 0.593 | 1 | 0.441 |
| **GLOBAL** | 30.037 | 24 | 0.184 |

**Table S4** Baseline characteristics of patients with dysphagia according to pneumonia-related mortality status.

| **Variables** | **Total** | **Alive** | **Death** | ***P*-value** |
| --- | --- | --- | --- | --- |
|  | **(n = 247)** | **(n = 204)** | **(n = 43)** |  |
| **CUN-BAE, Mean ± SD** | 27.4 ± 7.5 | 26.6 ± 7.4 | 30.8 ± 7.0 | ***< 0.001*** |
| **ECORE-BF, Mean ± SD** | 217.7 ± 9.0 | 217.0 ± 8.9 | 221.0 ± 8.6 | ***0.007*** |
| **Deurenberg, Mean ± SD** | 30.2 ± 7.0 | 29.5 ± 6.9 | 33.2 ± 6.7 | ***0.002*** |
| **Demographic** |  |  |  |  |
| Age (years), Mean ± SD | 83.0 ± 9.3 | 83.1 ± 9.5 | 82.4 ± 8.4 | 0.644 |
| Gender（Male）, n (%) | 151 (61.1) | 138 (67.6) | 13 (30.2) | ***< 0.001*** |
| **Nutritional parameters** |  |  |  |  |
| BMI, Mean ± SD | 19.2 ± 3.3 | 19.3 ± 3.4 | 19.1 ± 3.3 | 0.753 |
| TPN, n (%) | 67 (27.1) | 59 (28.9) | 8 (18.6) | 0.167 |
| PEG, n (%) | 180 (72.9) | 145 (71.1) | 35 (81.4) | 0.167 |
| NT.CVC, n (%) | 22 ( 8.9) | 19 (9.3) | 3 (7) | 0.775 |
| Nutrient intake, Kcal/day | 917.7 ± 187.5 | 963.9 ± 145.8 | 878.0 ± 209.5 | ***< 0.001*** |
| **Comorbidities** |  |  |  |  |
| CVD, n (%) | 132 (53.4) | 119 (58.3) | 13 (30.2) | ***< 0.001*** |
| Severe dementia, n (%) | 99 (40.1) | 75 (36.8) | 24 (55.8) | ***0.021*** |
| NMD, n (%) | 14 ( 5.7) | 10 (4.9) | 4 (9.3) | 0.275 |
| Asp, n (%) | 92 (37.2) | 61 (29.9) | 31 (72.1) | ***< 0.001*** |
| IHD, n (%) | 44 (17.8) | 34 (16.7) | 10 (23.3) | 0.305 |
| CHF, n (%) | 102 (41.3) | 83 (40.7) | 19 (44.2) | 0.672 |
| CPD, n (%) | 18 ( 7.3) | 14 (6.9) | 4 (9.3) | 0.528 |
| CLD, n (%) | 15 ( 6.1) | 11 (5.4) | 4 (9.3) | 0.305 |
| CKD, n (%) | 52 (21.1) | 42 (20.6) | 10 (23.3) | 0.697 |
| **Lab biomarkers** |  |  |  |  |
| ALB (g/dl), Mean ± SD | 3.1 ± 0.6 | 3.2 ± 0.6 | 2.9 ± 0.6 | ***0.030*** |
| TLC (10^9/L), Mean ± SD | 1.3 ± 0.7 | 1.4 ± 0.7 | 1.1 ± 0.5 | ***0.005*** |
| TC (mg/dl), Mean ± SD | 156.1 ± 40.2 | 158.3 ± 38.2 | 145.9 ± 47.7 | 0.071 |
| Hemoglobin (g/dl), Mean ± SD | 11.0 ± 2.0 | 11.1 ± 2.0 | 10.5 ± 2.1 | 0.116 |
| CRP (mg/l), Median (IQR) | 1.0 (0.3, 3.2) | 0.9 (0.3, 2.6) | 1.5 (0.4, 4.3) | 0.093 |
| **Outcomes** |  |  |  |  |
| Oral intake recovery, n (%) | 14 ( 5.7) | 14 (6.9) | 0 (0) | 0.138 |
| Discharge to home, n (%) | 38 (15.4) | 33 (16.2) | 5 (11.6) | 0.452 |
| Severe sepsis, n (%) | 29 (11.9) | 28 (13.9) | 1 (2.4) | 0.036 |

**Table S5** Association between nutritional indicators related to BMI index and sepsis-cause mortality across multivariable models.

|  | **Total** | **Event** | **Model 1** | |  | **Model 2** | |  | **Model 3** | |
| --- | --- | --- | --- | --- | --- | --- | --- | --- | --- | --- |
|  |  |  | **HR (95% CI)** | ***P* value** |  | **HR (95% CI)** | ***P* value** |  | **HR (95% CI)** | ***P* value** |
| **CUN-BAE** | 247 | 20 (8.1) | 1.03 (0.97~1.09) | 0.371 |  | 1.02 (0.96~1.08) | 0.502 |  | 1.06 (0.98~1.15) | 0.168 |
| T1 | 82 | 9 (11) | 1.0 [Ref] | |  | 1.0 [Ref] | |  | 1.0 [Ref] | |
| T2 | 82 | 3 (3.7) | 0.36 (0.10~1.33) | 0.126 |  | 0.34 (0.09~1.28) | 0.111 |  | 0.25 (0.05~1.20) | 0.084 |
| T3 | 83 | 8 (9.6) | 1.27 (0.48~3.33) | 0.633 |  | 1.20 (0.45~3.22) | 0.719 |  | 1.97 (0.48~8.10) | 0.347 |
| *P-*trend | 247 | 20 (8.1) | 1.10 (0.63~1.90) | 0.741 |  | 1.06 (0.60~1.85) | 0.843 |  | 1.21 (0.57~2.53) | 0.622 |
| **ECORE-BF** | 247 | 20 (8.1) | 1.01 (0.96~1.06) | 0.724 |  | 1 (0.96~1.06) | 0.863 |  | 1.03 (0.97~1.1) | 0.381 |
| T1 | 82 | 9 (11) | 1.0 [Ref] | |  | 1.0 [Ref] | |  | 1.0 [Ref] | |
| T2 | 82 | 2 (2.4) | 0.25 (0.05~1.15) | 0.075 |  | 0.26 (0.06~1.21) | 0.086 |  | 0.25 (0.04~1.47) | 0.125 |
| T3 | 83 | 9 (10.8) | 1.32 (0.52~3.36) | 0.556 |  | 1.37 (0.53~3.55) | 0.517 |  | 3.16 (0.83~12.01) | 0.091 |
| *P-*trend | 247 | 20 (8.1) | 1.15 (0.67~1.97) | 0.621 |  | 1.16 (0.67~2.00) | 0.596 |  | 1.54 (0.76~3.12) | 0.232 |
| **Deurenberg** | 247 | 20 (8.1) | 1.03 (0.97~1.1) | 0.306 |  | 1.03 (0.96~1.09) | 0.434 |  | 1.05 (0.97~1.14) | 0.224 |
| T1 | 82 | 8 (9.8) | 1.0 [Ref] | |  | 1.0 [Ref] | |  | 1.0 [Ref] | |
| T2 | 82 | 3 (3.7) | 0.43 (0.11~1.62) | 0.213 |  | 0.38 (0.10~1.43) | 0.153 |  | 0.25 (0.06~1.15) | 0.075 |
| T3 | 83 | 9 (10.8) | 1.69 (0.64~4.46) | 0.287 |  | 1.54 (0.57~4.15) | 0.396 |  | 2.80 (0.66~11.77) | 0.161 |
| *P-*trend | 247 | 20 (8.1) | 1.32 (0.76~2.28) | 0.326 |  | 1.25 (0.71~2.20) | 0.442 |  | 1.43 (0.66~3.10) | 0.366 |
| Model 1: no covariates were adjusted Model 2: CVD, Severe dementia, NMD, IHD were adjusted Model 3: CVD, Severe dementia, NMD, IHD, Asp, CHF, CLD, CPD, CKD, NT.CVC, PEG, oral intake recovery, CRP, ALB, TLC, TC, Nutrient intake, Hemoglobin. | | | | | | | | | | |
|  |  |  |  |  |  |  |  |  |  |  |
|  |  |  |  |  |  |  |  |  |  |  |
|  |  |  |  |  |  |  |  |  |  |  |

**Table S6** The confidence Intervals and Area Under the Curve (AUC) Values for all-cause mortality.

|  | AUC at 1 year | | |  | AUC at 2 years | | |  | AUC at 3 years | | |
| --- | --- | --- | --- | --- | --- | --- | --- | --- | --- | --- | --- |
| Variable | AUC | CI_Lower | CI_Upper |  | AUC | CI_Lower | CI_Upper |  | AUC | CI_Lower | CI_Upper |
| CUN-BAE | 0.633 | 0.556 | 0.709 |  | 0.685 | 0.595 | 0.775 |  | 0.728 | 0.599 | 0.858 |
| ECORE-BF | 0.594 | 0.516 | 0.671 |  | 0.638 | 0.546 | 0.731 |  | 0.676 | 0.536 | 0.815 |
| Deurenberg | 0.639 | 0.564 | 0.715 |  | 0.695 | 0.606 | 0.784 |  | 0.730 | 0.599 | 0.860 |

*Abbreviations:* ROC: receiver operating characteristic, AUC: area under the curve, CUN-BAE, Clínica Universidad de Navarra-Body Adiposity Estimator; ECORE-BF, Córdoba Equation for Estimation of Body Fat. CI: condidence interval. CI_Lower: confidence interval lower limit. CI_Upper: confidence interval upper limit. CVD: cardiovascular disease

**Table S7** The confidence Intervals and Area Under the Curve (AUC) Values for pneumonia-cause mortality.

|  | AUC at 1 year | | |  | AUC at 2 years | | |  | AUC at 3 years | | |
| --- | --- | --- | --- | --- | --- | --- | --- | --- | --- | --- | --- |
| Variable | AUC | CI_Lower | CI_Upper |  | AUC | CI_Lower | CI_Upper |  | AUC | CI_Lower | CI_Upper |
| CUN-BAE | 0.718 | 0.617 | 0.820 |  | 0.766 | 0.663 | 0.868 |  | 0.809 | 0.679 | 0.939 |
| ECORE-BF | 0.671 | 0.565 | 0.781 |  | 0.725 | 0.616 | 0.835 |  | 0.755 | 0.608 | 0.903 |
| Deurenberg | 0.713 | 0.613 | 0.814 |  | 0.768 | 0.667 | 0.870 |  | 00.796 | 0.661 | 0.932 |

*Abbreviations:* ROC: receiver operating characteristic, AUC: area under the curve, CUN-BAE, Clínica Universidad de Navarra-Body Adiposity Estimator; ECORE-BF, Córdoba Equation for Estimation of Body Fat. CI: condidence interval. CI_Lower: confidence interval lower limit. CI_Upper: confidence interval upper limit. CVD: cardiovascular disease

**Table S8** C-Index values of nutritional indicators related to BMI for all-cause mortality.

| **Time (Years)** | **CUN-BAE** | **ECORE-BF** | **Deurenberg** |
| --- | --- | --- | --- |
| 1 | 0.601 | 0.565 | 0.605 |
| 1.5 | 0.607 | 0.572 | 0.611 |
| 2 | 0.605 | 0.569 | 0.609 |
| 2.5 | 0.600 | 0.565 | 0.605 |
| 3 | 0.603 | 0.567 | 0.607 |
| 3.5 | 0.601 | 0.565 | 0.605 |
| 4 | 0.320 | 0.301 | 0.322 |

*Abbrevaitions:* C-index: concordance index; CUN-BAE, Clínica Universidad de Navarra-Body Adiposity Estimator; ECORE-BF, Córdoba Equation for Estimation of Body Fat.

**Table S9** C-Index values of nutritional indicators related to BMI for pneumonia-cause mortality.

| **Time （years）** | **CUN-BAE** | **ECORE-BF** | **Deurenberg** |
| --- | --- | --- | --- |
| 1 | 0.601 | 0.565 | 0.605 |
| 1.5 | 0.607 | 0.572 | 0.611 |
| 2 | 0.605 | 0.569 | 0.609 |
| 2.5 | 0.600 | 0.565 | 0.605 |
| 3 | 0.603 | 0.567 | 0.607 |
| 3.5 | 0.601 | 0.565 | 0.605 |
| 4 | 0.320 | 0.301 | 0.322 |

*Abbrevaitions:* C-index: concordance index; CUN-BAE, Clínica Universidad de Navarra-Body Adiposity Estimator; ECORE-BF, Córdoba Equation for Estimation of Body Fat.

**Table S10** Incremental Value of nutritional indicators related to BMI in all-cause mortality Prediction

| Event | NRI (95% CI) | NRI P value | IDI (95% CI) | IDI P value |
| --- | --- | --- | --- | --- |
| Basic model | Ref | - | Ref | - |
| Basic model + CUN-BAE | 0.468 (0.040-0.879) | <0.05 | 0.028 (0.001-0.076) | 0.040 |
| Basic model + ECORE-BF | 0.380 (-0.031-0.700) | >0.05 | 0.014 (-0.004-0.048) | 0.159 |
| Basic model + Deurenberg | 0.475 (0.009-0.857) | <0.05 | 0.023 (0.000-0.072) | 0.060 |

Basic Model: This model includes variables CVD, Severe dementia, NMD, IHD, Asp, CHF, CLD, CPD, CKD, NT.CVC, PEG, oral intake recovery. Abbreviation: CVD, cerebrovascular diseases; NMD, neuromuscular diseases; Asp, previous history of aspiration pneumonia; IHD, ischemic heart diseases; CHF, Chronic Heart Failure; CPD, Chronic Pulmonary Disease, CLD, Chronic Liver Diseases; CKD, Chronic Kidney Diseases; ALB, serum albumin; TLC, Total Lymphocyte Count; TC, Total Cholesterol; CRP, C-reactive Protein; NT.CVC, Non-tunneled Central Venous Catheters; PEG, Percutaneous Endoscopic Gastrostomy; TPN, Total Parenteral Nutrition; BMI, Body Mass Index; CUN-BAE, Clínica Universidad de Navarra-Body Adiposity Estimator; ECORE-BF, Córdoba Equation for Estimation of Body Fat. CI confidence interval; Ref reference.

**Table S11** Incremental Value of nutritional indicators related to BMI in pneumonia-cause mortality Prediction

| Event | NRI (95% CI) | NRI P value | IDI (95% CI) | IDI P value |
| --- | --- | --- | --- | --- |
| Basic model | Ref | - | Ref | - |
| Basic model + CUN-BAE | 0.520 (-0.074-1.256) | >0.05 | 0.030 (-0.003-0.125) | >0.05 |
| Basic model + ECORE-BF | 0.401 (-0.308-1.142) | >0.05 | 0.016 (-0.003-0.098) | >0.05 |
| Basic model + Deurenberg | 0.436 (-0.096-1.177) | >0.05 | 0.020 (-0.003-0.108) | >0.05 |

Basic Model: This model includes variables CVD, Severe dementia, NMD, IHD, Asp, CHF, CLD, CPD, CKD, NT.CVC, PEG, oral intake recovery. Abbreviation: CVD, cerebrovascular diseases; NMD, neuromuscular diseases; Asp, previous history of aspiration pneumonia; IHD, ischemic heart diseases; CHF, Chronic Heart Failure; CPD, Chronic Pulmonary Disease, CLD, Chronic Liver Diseases; CKD, Chronic Kidney Diseases; ALB, serum albumin; TLC, Total Lymphocyte Count; TC, Total Cholesterol; CRP, C-reactive Protein; NT.CVC, Non-tunneled Central Venous Catheters; PEG, Percutaneous Endoscopic Gastrostomy; TPN, Total Parenteral Nutrition; BMI, Body Mass Index; CUN-BAE, Clínica Universidad de Navarra-Body Adiposity Estimator; ECORE-BF, Córdoba Equation for Estimation of Body Fat. CI confidence interval; Ref reference.

**Table S12** Association Between nutritional indicators related to BMI and all-cause mortality Across Multivariable Models after MICE.

|  | **Total** | **Event** | **Model 1** | |  | **Model 2** | |  | **Model 3** | |
| --- | --- | --- | --- | --- | --- | --- | --- | --- | --- | --- |
|  |  |  | **HR (95% CI)** | ***P* value** |  | **HR (95% CI)** | ***P* value** |  | **HR (95% CI)** | ***P* value** |
| **CUN-BAE** | 247 | 133 (53.8) | 1.05 (1.03~1.07) | <0.001 |  | 1.05 (1.02~1.07) | <0.001 |  | 1.06 (1.03~1.09) | <0.001 |
| T1 | 82 | 37 (45.1) | 1.0 [Ref] | |  | 1.0 [Ref] | |  | 1.0 [Ref] | |
| T2 | 82 | 37 (45.1) | 1.09 (0.69~1.72) | 0.718 |  | 1.12 (0.71~1.77) | 0.633 |  | 1.12 (0.69~1.83) | 0.648 |
| T3 | 83 | 59 (71.1) | 2.18 (1.44~3.30) | <0.001 |  | 2.15 (1.40~3.31) | <0.001 |  | 2.35 (1.42~3.89) | 0.001 |
| *P-*trend | 247 | 133 (53.8) | 1.51 (1.22~1.87) | <0.001 |  | 1.48 (1.19~1.85) | <0.001 |  | 1.53 (1.18~1.99) | 0.001 |
| **ECORE-BF** | 247 | 133 (53.8) | 1.03 (1.01~1.05) | 0.002 |  | 1.03 (1.01~1.05) | 0.004 |  | 1.03 (1.01~1.06) | 0.004 |
| T1 | 82 | 40 (48.8) | 1.0 [Ref] | |  | 1.0 [Ref] | |  | 1.0 [Ref] | |
| T2 | 82 | 36 (43.9) | 1.00 (0.64~1.57) | 0.990 |  | 1.06 (0.68~1.67) | 0.796 |  | 1.46 (0.90~2.36) | 0.124 |
| T3 | 83 | 57 (68.7) | 1.83 (1.22~2.74) | 0.004 |  | 1.90 (1.25~2.89) | 0.003 |  | 1.96 (1.20~3.20) | 0.007 |
| *P-*trend | 247 | 133 (53.8) | 1.37 (1.11~1.70) | 0.003 |  | 1.39 (1.12~1.73) | 0.003 |  | 1.40 (1.10~1.79) | 0.007 |
| **Deurenberg** | 247 | 133 (53.8) | 1.06 (1.03~1.08) | <0.001 |  | 1.05 (1.03~1.08) | <0.001 |  | 1.06 (1.03~1.09) | <0.001 |
| T1 | 82 | 35 (42.7) | 1.0 [Ref] | |  | 1.0 [Ref] | |  | 1.0 [Ref] | |
| T2 | 82 | 38 (46.3) | 1.24 (0.78~1.96) | 0.363 |  | 1.16 (0.73~1.84) | 0.527 |  | 1.05 (0.64~1.73) | 0.833 |
| T3 | 83 | 60 (72.3) | 2.46 (1.61~3.75) | <0.001 |  | 2.31 (1.49~3.58) | <0.001 |  | 2.31 (1.38~3.87) | 0.001 |
| *P-*trend | 247 | 133 (53.8) | 1.60 (1.29~1.99) | <0.001 |  | 1.55 (1.23~1.94) | <0.001 |  | 1.53 (1.17~2.00) | 0.002 |
| Model 1: no covariates were adjusted Model 2: CVD, Severe dementia, NMD, IHD were adjusted Model 3: CVD, Severe dementia, NMD, IHD, Asp, CHF, CLD, CPD, CKD, NT.CVC, PEG, oral intake recovery, CRP, ALB, TLC, TC, Nutrient intake, Hemoglobin. | | | | | | | | | | |
|  |  |  |  |  |  |  |  |  |  |  |
|  |  |  |  |  |  |  |  |  |  |  |
|  |  |  |  |  |  |  |  |  |  |  |

**Table S13** Association Between nutritional indicators related to BMI and pneumonia-cause mortality Across Multivariable Models after MICE.

|  | **Total** | **Event** | **Model 1** | |  | **Model 2** | |  | **Model 3** | |
| --- | --- | --- | --- | --- | --- | --- | --- | --- | --- | --- |
|  |  |  | **HR (95% CI)** | P **value** |  | **HR (95% CI)** | P **value** |  | **HR (95% CI)** | P **value** |
| **CUN-BAE** | 247 | 43 (17.4) | 1.08 (1.04~1.12) | <0.001 |  | 1.09 (1.04~1.13) | <0.001 |  | 1.09 (1.04~1.15) | 0.001 |
| T1 | 82 | 8 (9.8) | 1.0 [Ref] | |  | 1.0 [Ref] | |  | 1.0 [Ref] | |
| T2 | 82 | 12 (14.6) | 1.66 (0.68~4.05) | 0.269 |  | 1.82 (0.74~4.49) | 0.195 |  | 2.62 (1.01~6.80) | 0.048 |
| T3 | 83 | 23 (27.7) | 3.84 (1.71~8.62) | 0.001 |  | 4.63 (2.02~10.63) | <0.001 |  | 4.18 (1.68~10.45) | 0.002 |
| *P-*trend | 247 | 43 (17.4) | 2.02 (1.35~3.01) | 0.001 |  | 2.21 (1.46~3.34) | <0.001 |  | 2.00 (1.29~3.11) | 0.002 |
| **ECORE-BF** | 247 | 43 (17.4) | 1.05 (1.02~1.09) | 0.002 |  | 1.06 (1.02~1.1) | 0.001 |  | 1.06 (1.02~1.1) | 0.004 |
| T1 | 82 | 9 (11) | 1.0 [Ref] | |  | 1.0 [Ref] | |  | 1.0 [Ref] | |
| T2 | 82 | 13 (15.9) | 1.62 (0.69~3.79) | 0.267 |  | 1.83 (0.78~4.33) | 0.167 |  | 2.63 (1.07~6.46) | 0.034 |
| T3 | 83 | 21 (25.3) | 2.94 (1.34~6.45) | 0.007 |  | 3.62 (1.62~8.08) | 0.002 |  | 3.28 (1.33~8.12) | 0.010 |
| *P-*trend | 247 | 43 (17.4) | 1.73 (1.18~2.54) | 0.005 |  | 1.91 (1.29~2.84) | 0.001 |  | 1.77 (1.15~2.73) | 0.010 |
| **Deurenberg** | 247 | 43 (17.4) | 1.08 (1.04~1.13) | <0.001 |  | 1.08 (1.04~1.13) | <0.001 |  | 1.08 (1.03~1.14) | 0.002 |
| T1 | 82 | 7 (8.5) | 1.0 [Ref] | |  | 1.0 [Ref] | |  | 1.0 [Ref] | |
| T2 | 82 | 13 (15.9) | 2.14 (0.85~5.37) | 0.105 |  | 2.06 (0.81~5.19) | 0.127 |  | 2.59 (0.96~6.99) | 0.060 |
| T3 | 83 | 23 (27.7) | 4.60 (1.96~10.79) | <0.001 |  | 5.12 (2.14~12.25) | <0.001 |  | 4.29 (1.62~11.33) | 0.003 |
| *P-*trend | 247 | 43 (17.4) | 2.15 (1.43~3.21) | <0.001 |  | 2.31 (1.51~3.52) | <0.001 |  | 2.01 (1.27~3.19) | 0.003 |
| Model 1: no covariates were adjusted Model 2: CVD, Severe dementia, NMD, IHD were adjusted Model 3: CVD, Severe dementia, NMD, IHD, Asp, CHF, CLD, CPD, CKD, NT.CVC, PEG, oral intake recovery, CRP, ALB, TLC, TC, Nutrient intake, Hemoglobin. | | | | | | | | | | |
|  |  |  |  |  |  |  |  |  |  |  |
|  |  |  |  |  |  |  |  |  |  |  |
|  |  |  |  |  |  |  |  |  |  |  |

**Table S14** Associations nutritional indicators related to BMI and all-cause mortality in patients with survival of more than 30 days.

|  | **Total** | **Event** | **Model 1** | |  | **Model 2** | |  | **Model 3** | |
| --- | --- | --- | --- | --- | --- | --- | --- | --- | --- | --- |
|  |  |  | **HR (95% CI)** | ***P* value** |  | **HR (95% CI)** | ***P* value** |  | **HR (95% CI)** | ***P* value** |
| **CUN-BAE** | 227 | 115 (50.7) | 1.05 (1.03~1.08) | <0.001 |  | 1.06 (1.03~1.08) | <0.001 |  | 1.07 (1.04~1.11) | <0.001 |
| T1 | 76 | 31 (40.8) | 1.0 [Ref] | |  | 1.0 [Ref] | |  | 1.0 [Ref] | |
| T2 | 75 | 31 (41.3) | 1.08 (0.66~1.78) | 0.753 |  | 1.11 (0.67~1.84) | 0.679 |  | 1.19 (0.69~2.06) | 0.530 |
| T3 | 76 | 53 (69.7) | 2.52 (1.61~3.94) | <0.001 |  | 2.76 (1.75~4.37) | <0.001 |  | 2.80 (1.59~4.92) | <0.001 |
| *P-*trend | 227 | 115 (50.7) | 1.64 (1.29~2.07) | <0.001 |  | 1.71 (1.35~2.18) | <0.001 |  | 1.67 (1.25~2.23) | 0.001 |
| **ECORE-BF** | 227 | 115 (50.7) | 1.03 (1.01~1.06) | 0.002 |  | 1.04 (1.02~1.06) | <0.001 |  | 1.05 (1.02~1.07) | 0.001 |
| T1 | 76 | 34 (44.7) | 1.0 [Ref] | |  | 1.0 [Ref] | |  | 1.0 [Ref] | |
| T2 | 75 | 30 (40) | 0.98 (0.60~1.60) | 0.931 |  | 1.03 (0.63~1.69) | 0.901 |  | 1.29 (0.75~2.22) | 0.362 |
| T3 | 76 | 51 (67.1) | 2.06 (1.33~3.18) | 0.001 |  | 2.31 (1.48~3.59) | <0.001 |  | 2.70 (1.55~4.70) | <0.001 |
| *P-*trend | 227 | 115 (50.7) | 1.47 (1.17~1.85) | 0.001 |  | 1.55 (1.23~1.96) | <0.001 |  | 1.64 (1.23~2.17) | 0.001 |
| **Deurenberg** | 227 | 115 (50.7) | 1.06 (1.04~1.09) | <0.001 |  | 1.06 (1.04~1.09) | <0.001 |  | 1.07 (1.04~1.11) | <0.001 |
| T1 | 76 | 29 (38.2) | 1.0 [Ref] | |  | 1.0 [Ref] | |  | 1.0 [Ref] | |
| T2 | 75 | 33 (44) | 1.29 (0.78~2.13) | 0.315 |  | 1.23 (0.75~2.04) | 0.415 |  | 1.19 (0.69~2.05) | 0.533 |
| T3 | 76 | 53 (69.7) | 2.77 (1.75~4.37) | <0.001 |  | 2.95 (1.85~4.71) | <0.001 |  | 2.94 (1.64~5.27) | <0.001 |
| *P-*trend | 227 | 115 (50.7) | 1.70 (1.35~2.16) | <0.001 |  | 1.77 (1.39~2.26) | <0.001 |  | 1.72 (1.27~2.32) | <0.001 |
| Model 1: no covariates were adjusted Model 2: CVD, Severe dementia, NMD, IHD were adjusted Model 3: CVD, Severe dementia, NMD, IHD, Asp, CHF, CLD, CPD, CKD, NT.CVC, PEG, oral intake recovery, CRP, ALB, TLC, TC, Nutrient intake, Hemoglobin. | | | | | | | | | | |
|  |  |  |  |  |  |  |  |  |  |  |
|  |  |  |  |  |  |  |  |  |  |  |
|  |  |  |  |  |  |  |  |  |  |  |

**Table S15** Associations nutritional indicators related to BMI and pneumonia-cause in patients with survival of more than 30 days.

|  | **Total** | **Event** | **Model 1** | |  | **Model 2** | |  | **Model 3** | |
| --- | --- | --- | --- | --- | --- | --- | --- | --- | --- | --- |
|  |  |  | **HR (95% CI)** | ***P* value** |  | **HR (95% CI)** | ***P* value** |  | **HR (95% CI)** | ***P* value** |
| **CUN-BAE** | 227 | 38 (16.7) | 1.09 (1.05~1.14) | <0.001 |  | 1.08 (1.03~1.13) | 0.001 |  | 1.12 (1.06~1.19) | <0.001 |
| T1 | 76 | 6 (7.9) | 1.0 [Ref] | |  | 1.0 [Ref] | |  | 1.0 [Ref] | |
| T2 | 75 | 10 (13.3) | 1.83 (0.67~5.04) | 0.241 |  | 2.19 (0.79~6.09) | 0.132 |  | 3.75 (1.21~11.66) | 0.022 |
| T3 | 76 | 22 (28.9) | 5.22 (2.11~12.95) | <0.001 |  | 4.73 (1.86~12.05) | 0.001 |  | 6.89 (2.32~20.46) | 0.001 |
| *P-*trend | 227 | 38 (16.7) | 2.4 (1.54~3.74) | <0.001 |  | 2.17 (1.39~3.40) | 0.001 |  | 2.50 (1.51~4.16) | <0.001 |
| **ECORE-BF** | 227 | 38 (16.7) | 1.06 (1.03~1.10) | 0.001 |  | 1.06 (1.02~1.10) | 0.002 |  | 1.09 (1.04~1.14) | <0.001 |
| T1 | 76 | 7 (9.2) | 1.0 [Ref] | |  | 1.0 [Ref] | |  | 1.0 [Ref] | |
| T2 | 75 | 11 (14.7) | 1.75 (0.68~4.51) | 0.248 |  | 2.08 (0.80~5.40) | 0.134 |  | 3.11 (1.10~8.82) | 0.032 |
| T3 | 76 | 20 (26.3) | 3.82 (1.61~9.07) | 0.002 |  | 3.59 (1.47~8.74) | 0.005 |  | 6.26 (2.11~18.54) | 0.001 |
| *P-*trend | 227 | 38 (16.7) | 1.99 (1.31~3.04) | 0.001 |  | 1.87 (1.22~2.86) | 0.004 |  | 2.44 (1.45~4.12) | 0.001 |
| **Deurenberg** | 227 | 38 (16.7) | 1.10 (1.05~1.15) | <0.001 |  | 1.08 (1.03~1.13) | 0.001 |  | 1.12 (1.05~1.18) | <0.001 |
| T1 | 76 | 5 (6.6) | 1.0 [Ref] | |  | 1.0 [Ref] | |  | 1.0 [Ref] | |
| T2 | 75 | 12 (16) | 2.75 (0.97~7.82) | 0.057 |  | 2.67 (0.94~7.60) | 0.066 |  | 4.45 (1.31~15.09) | 0.016 |
| T3 | 76 | 21 (27.6) | 6.13 (2.30~16.35) | <0.001 |  | 5.08 (1.86~13.88) | 0.002 |  | 9.30 (2.66~32.55) | <0.001 |
| *P-*trend | 227 | 38 (16.7) | 2.41 (1.55~3.75) | <0.001 |  | 2.17 (1.37~3.44) | 0.001 |  | 2.84 (1.61~5.02) | <0.001 |
| Model 1: no covariates were adjusted Model 2: CVD, Severe dementia, NMD, IHD were adjusted Model 3: CVD, Severe dementia, NMD, IHD, Asp, CHF, CLD, CPD, CKD, NT.CVC, PEG, oral intake recovery, CRP, ALB, TLC, TC, Nutrient intake, Hemoglobin. | | | | | | | | | | |
|  |  |  |  |  |  |  |  |  |  |  |
|  |  |  |  |  |  |  |  |  |  |  |
|  |  |  |  |  |  |  |  |  |  |  |

**Table S16** Association Between nutritional indicators related to BMI and all-cause mortality Across Additional adjusted for CFS in Multivariable Models.

|  | **Total** | **Event** | **Model 1** | |  | **Model 2** | |  | **Model 3** | |
| --- | --- | --- | --- | --- | --- | --- | --- | --- | --- | --- |
|  |  |  | **HR (95% CI)** | ***P* value** |  | **HR (95% CI)** | ***P* value** |  | **HR (95% CI)** | ***P* value** |
| **CUN-BAE** | 247 | 133 (53.8) | 1.05 (1.03~1.07) | <0.001 |  | 1.05 (1.02~1.07) | <0.001 |  | 1.05 (1.02~1.08) | <0.001 |
| T1 | 82 | 37 (45.1) | 1.0 [Ref] | |  | 1.0 [Ref] | |  | 1.0 [Ref] | |
| T2 | 82 | 37 (45.1) | 1.09 (0.69~1.72) | 0.718 |  | 1.12 (0.71~1.77) | 0.633 |  | 1.25 (0.76~2.05) | 0.383 |
| T3 | 83 | 59 (71.1) | 2.18 (1.44~3.30) | <0.001 |  | 2.15 (1.40~3.31) | <0.001 |  | 2.06 (1.23~3.46) | 0.006 |
| *P-*trend | 247 | 133 (53.8) | 1.51 (1.22~1.87) | <0.001 |  | 1.48 (1.19~1.85) | <0.001 |  | 1.43 (1.10~1.86) | 0.007 |
| **ECORE-BF** | 247 | 133 (53.8) | 1.03 (1.01~1.05) | 0.002 |  | 1.03 (1.01~1.05) | 0.004 |  | 1.03 (1.01~1.05) | 0.013 |
| T1 | 82 | 40 (48.8) | 1.0 [Ref] | |  | 1.0 [Ref] | |  | 1.0 [Ref] | |
| T2 | 82 | 36 (43.9) | 1.00 (0.64~1.57) | 0.990 |  | 1.06 (0.68~1.67) | 0.796 |  | 1.42 (0.87~2.32) | 0.162 |
| T3 | 83 | 57 (68.7) | 1.83 (1.22~2.74) | 0.004 |  | 1.90 (1.25~2.89) | 0.003 |  | 1.75 (1.06~2.90) | 0.029 |
| *P-*trend | 247 | 133 (53.8) | 1.37 (1.11~1.70) | 0.003 |  | 1.39 (1.12~1.73) | 0.003 |  | 1.33 (1.03~1.70) | 0.028 |
| **Deurenberg** | 247 | 133 (53.8) | 1.06 (1.03~1.08) | <0.001 |  | 1.05 (1.03~1.08) | <0.001 |  | 1.05 (1.02~1.08) | 0.002 |
| T1 | 82 | 35 (42.7) | 1.0 [Ref] | |  | 1.0 [Ref] | |  | 1.0 [Ref] | |
| T2 | 82 | 38 (46.3) | 1.24 (0.78~1.96) | 0.363 |  | 1.16 (0.73~1.84) | 0.527 |  | 1.13 (0.69~1.87) | 0.622 |
| T3 | 83 | 60 (72.3) | 2.46 (1.61~3.75) | <0.001 |  | 2.31 (1.49~3.58) | <0.001 |  | 2.00 (1.18~3.40) | 0.010 |
| *P-*trend | 247 | 133 (53.8) | 1.60 (1.29~1.99) | <0.001 |  | 1.55 (1.23~1.94) | <0.001 |  | 1.41 (1.08~1.85) | 0.012 |
| Model 1: no covariates were adjusted Model 2: CVD, Severe dementia, NMD, IHD were adjusted Model 3: CVD, Severe dementia, NMD, IHD, Asp, CHF, CLD, CPD, CKD, NT.CVC, PEG, oral intake recovery, CRP, ALB, TLC, TC, Nutrient intake, Hemoglobin, CFS. | | | | | | | | | | |
|  |  |  |  |  |  |  |  |  |  |  |
|  |  |  |  |  |  |  |  |  |  |  |
|  |  |  |  |  |  |  |  |  |  |  |

**Table S17** Association Between nutritional indicators related to BMI and pneumonia-cause mortality Across Additional adjusted for CFS in Multivariable Models.

|  | **Total** | **Event** | **Model 1** | |  | **Model 2** | |  | **Model 3** | |
| --- | --- | --- | --- | --- | --- | --- | --- | --- | --- | --- |
|  |  |  | **HR (95% CI)** | ***P* value** |  | **HR (95% CI)** | ***P* value** |  | **HR (95% CI)** | ***P* value** |
| **CUN-BAE** | 247 | 43 (17.4) | 1.08 (1.04~1.12) | <0.001 |  | 1.09 (1.04~1.13) | <0.001 |  | 1.09 (1.03~1.15) | 0.002 |
| T1 | 82 | 8 (9.8) | 1.0 [Ref] | |  | 1.0 [Ref] | |  | 1.0 [Ref] | |
| T2 | 82 | 12 (14.6) | 1.66 (0.68~4.05) | 0.269 |  | 1.82 (0.74~4.49) | 0.195 |  | 3.95 (1.43~10.93) | 0.008 |
| T3 | 83 | 23 (27.7) | 3.84 (1.71~8.62) | 0.001 |  | 4.63 (2.02~10.63) | <0.001 |  | 4.06 (1.54~10.74) | 0.005 |
| *P-*trend | 247 | 43 (17.4) | 2.02 (1.35~3.01) | 0.001 |  | 2.21 (1.46~3.34) | <0.001 |  | 1.92 (1.22~3.01) | 0.005 |
| **ECORE-BF** | 247 | 43 (17.4) | 1.05 (1.02~1.09) | 0.002 |  | 1.06 (1.02~1.1) | 0.001 |  | 1.06 (1.02~1.10) | 0.006 |
| T1 | 82 | 9 (11) | 1.0 [Ref] | |  | 1.0 [Ref] | |  | 1.0 [Ref] | |
| T2 | 82 | 13 (15.9) | 1.62 (0.69~3.79) | 0.267 |  | 1.83 (0.78~4.33) | 0.167 |  | 3.52 (1.34~9.05) | 0.009 |
| T3 | 83 | 21 (25.3) | 2.94 (1.34~6.45) | 0.007 |  | 3.62 (1.62~8.08) | 0.002 |  | 3.24 (1.24~8.48) | 0.016 |
| *P-*trend | 247 | 43 (17.4) | 1.73 (1.18~2.54) | 0.005 |  | 1.91 (1.29~2.84) | 0.001 |  | 1.75 (1.11~2.75) | 0.015 |
| **Deurenberg** | 247 | 43 (17.4) | 1.08 (1.04~1.13) | <0.001 |  | 1.08 (1.04~1.13) | <0.001 |  | 1.08 (1.03~1.14) | 0.004 |
| T1 | 82 | 7 (8.5) | 1.0 [Ref] | |  | 1.0 [Ref] | |  | 1.0 [Ref] | |
| T2 | 82 | 13 (15.9) | 2.14 (0.85~5.37) | 0.105 |  | 2.06 (0.81~5.19) | 0.127 |  | 3.76 (1.28~11.07) | 0.016 |
| T3 | 83 | 23 (27.7) | 4.60 (1.96~10.79) | <0.001 |  | 5.12 (2.14~12.25) | <0.001 |  | 4.18 (1.46~8.48) | 0.008 |
| *P-*trend | 247 | 43 (17.4) | 2.15 (1.43~3.21) | <0.001 |  | 2.31 (1.51~3.52) | <0.001 |  | 1.91 (1.19~3.08) | 0.008 |
| Model 1: no covariates were adjusted Model 2: CVD, Severe dementia, NMD, IHD were adjusted Model 3: CVD, Severe dementia, NMD, IHD, Asp, CHF, CLD, CPD, CKD, NT.CVC, PEG, oral intake recovery, CRP, ALB, TLC, TC, Nutrient intake, Hemoglobin. | | | | | | | | | | |
|  |  |  |  |  |  |  |  |  |  |  |
|  |  |  |  |  |  |  |  |  |  |  |
|  |  |  |  |  |  |  |  |  |  |  |


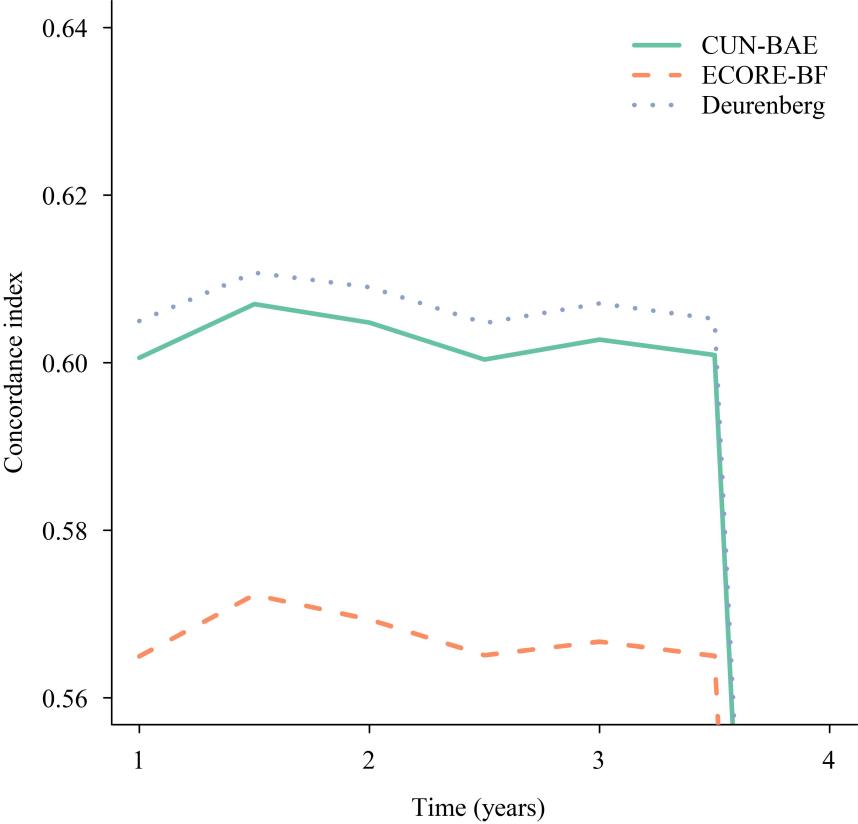


**Fig. S1** C-index evaluation for nutritional indicators related to BMI in forecasting all-cause mortality.

**
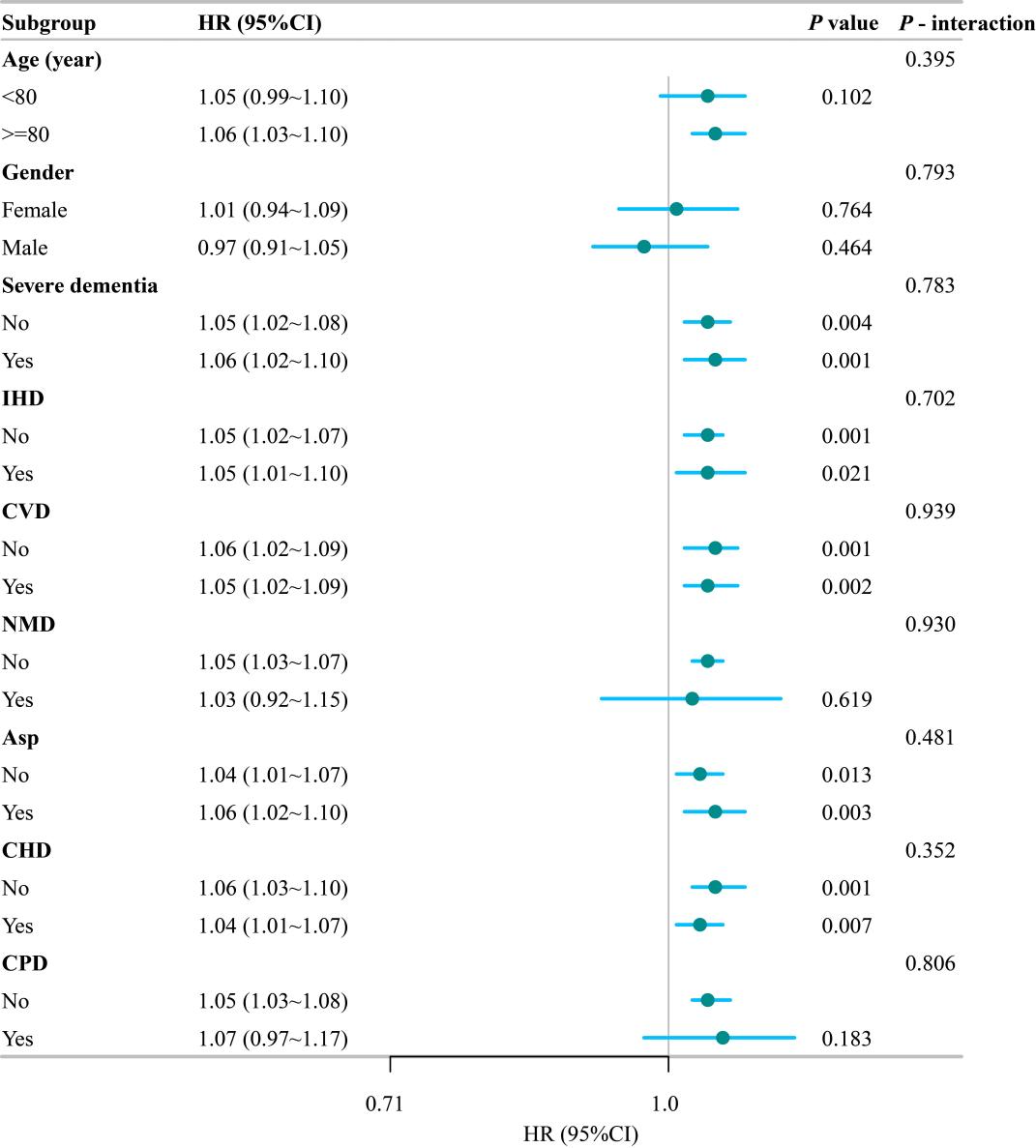
**

**Fig. S2** Subgroup analysis of the relationship between CUN-BAE and all-cause mortality.


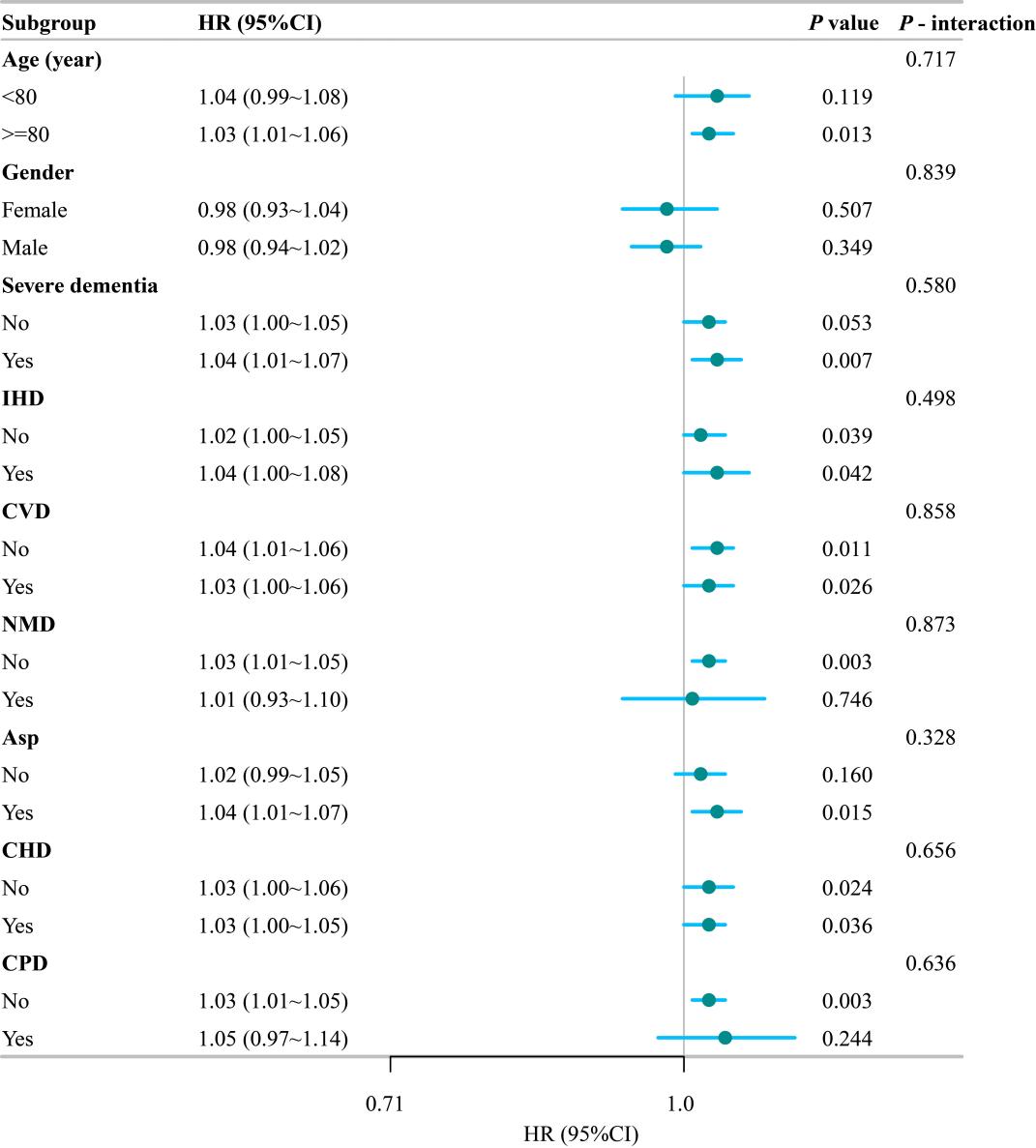


**Fig. S3** Subgroup analysis of the relationship between ECORE-BF and all-cause mortality.


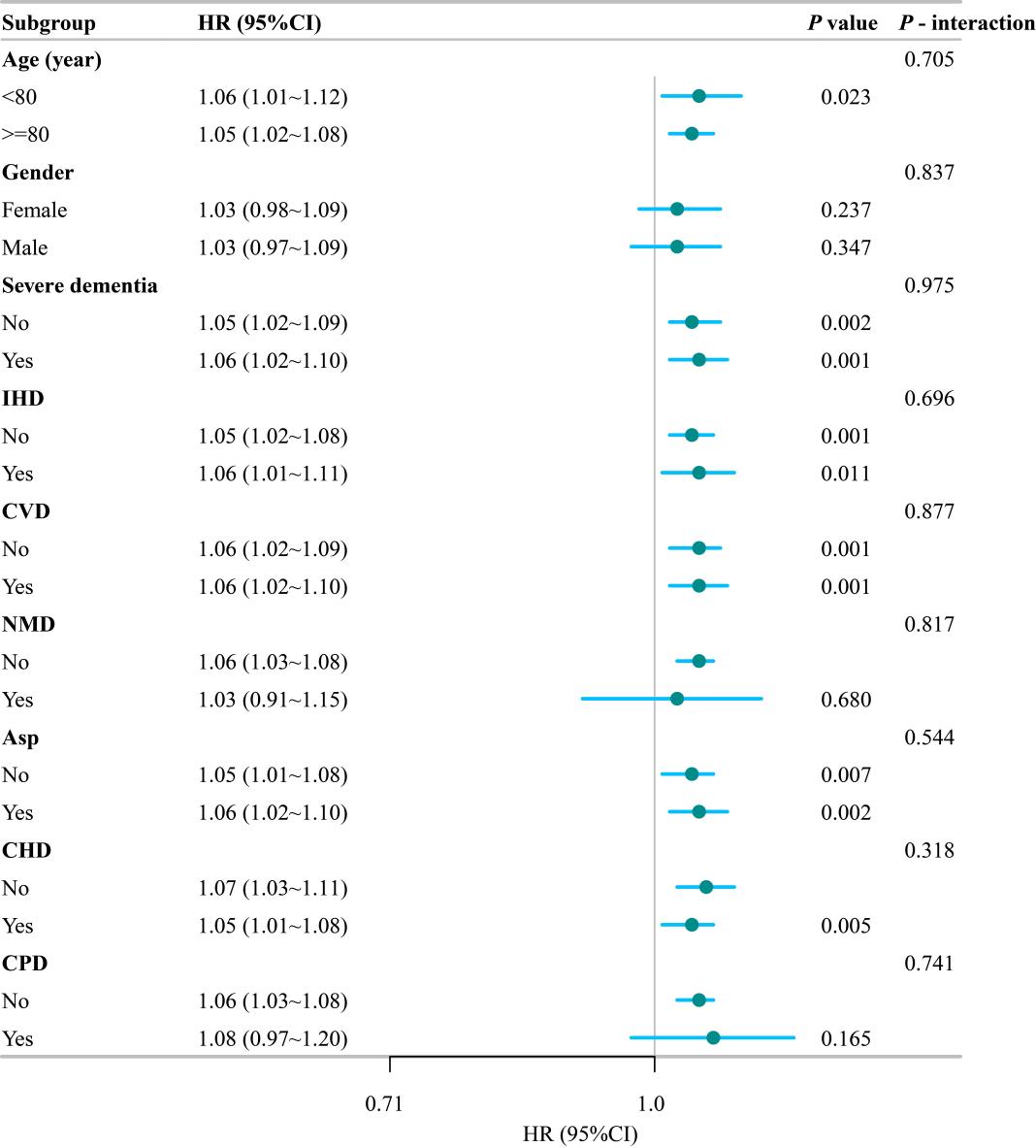


**Fig. S4** Subgroup analysis of the relationship between Deurenberg and all-cause mortality.


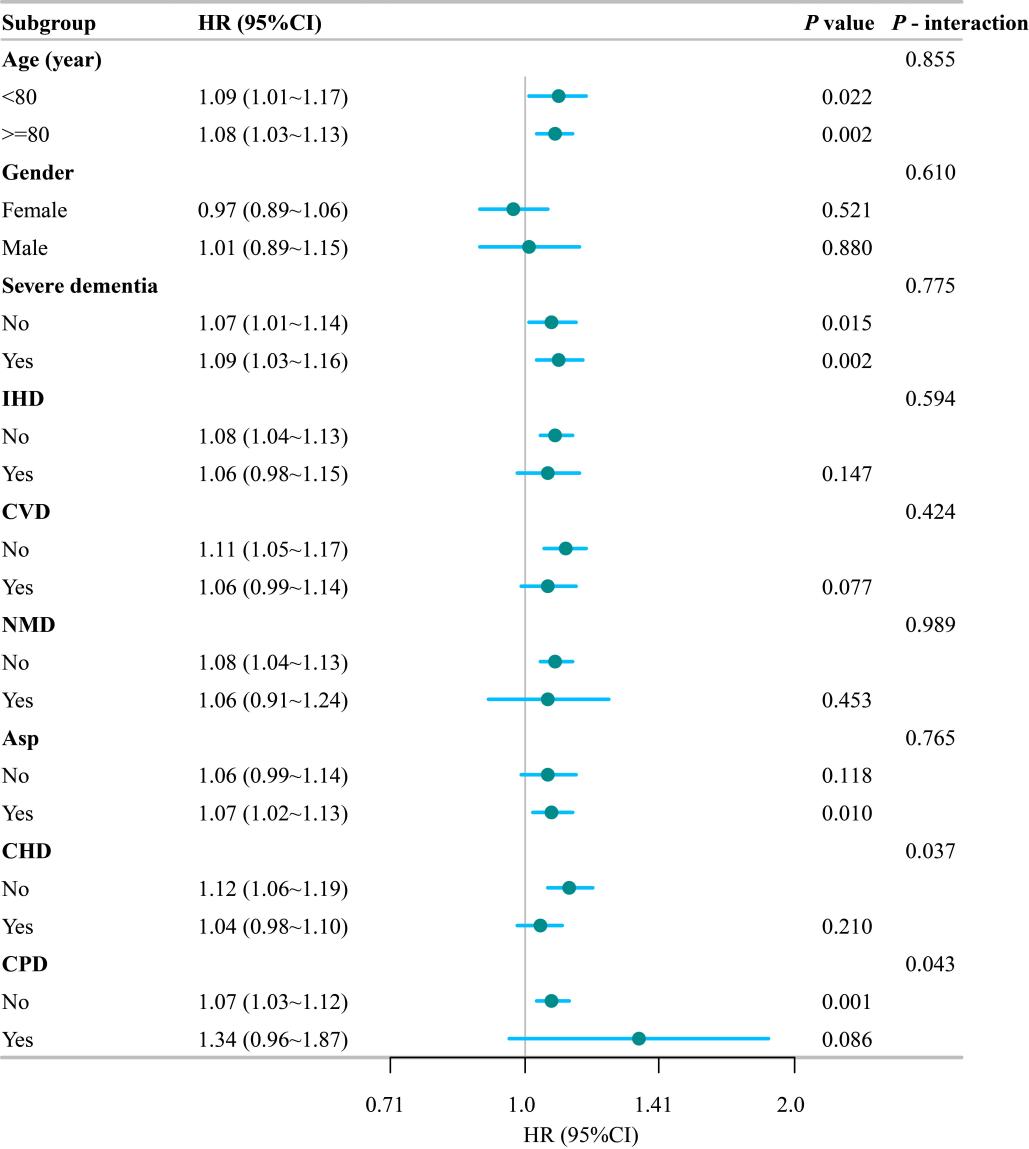


**Fig. S5** Subgroup analysis of the relationship between CUN-BAE and pneumonia-cause.


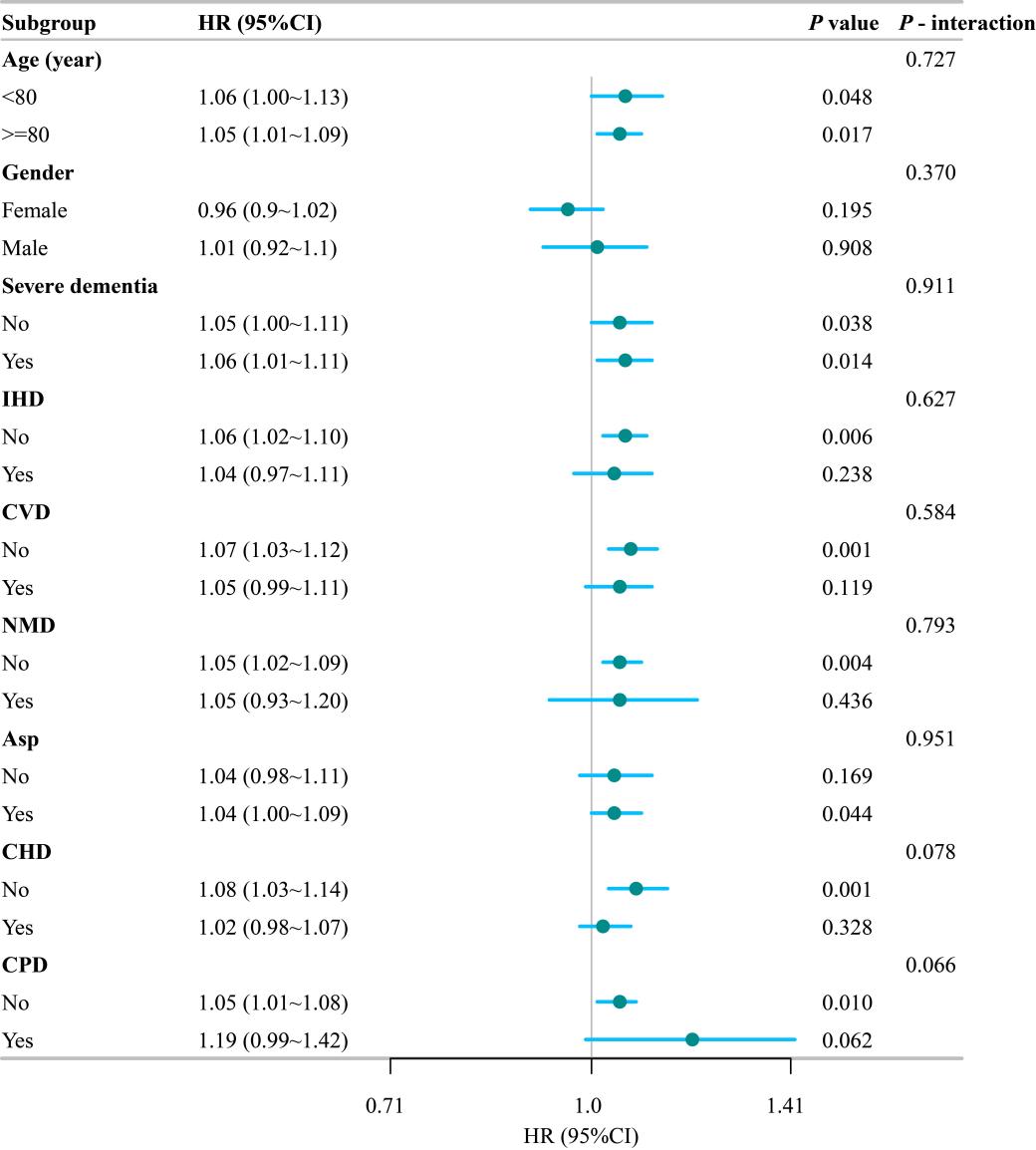


**Fig. S6** Subgroup analysis of the relationship between ECORE-BF and pneumonia-cause.


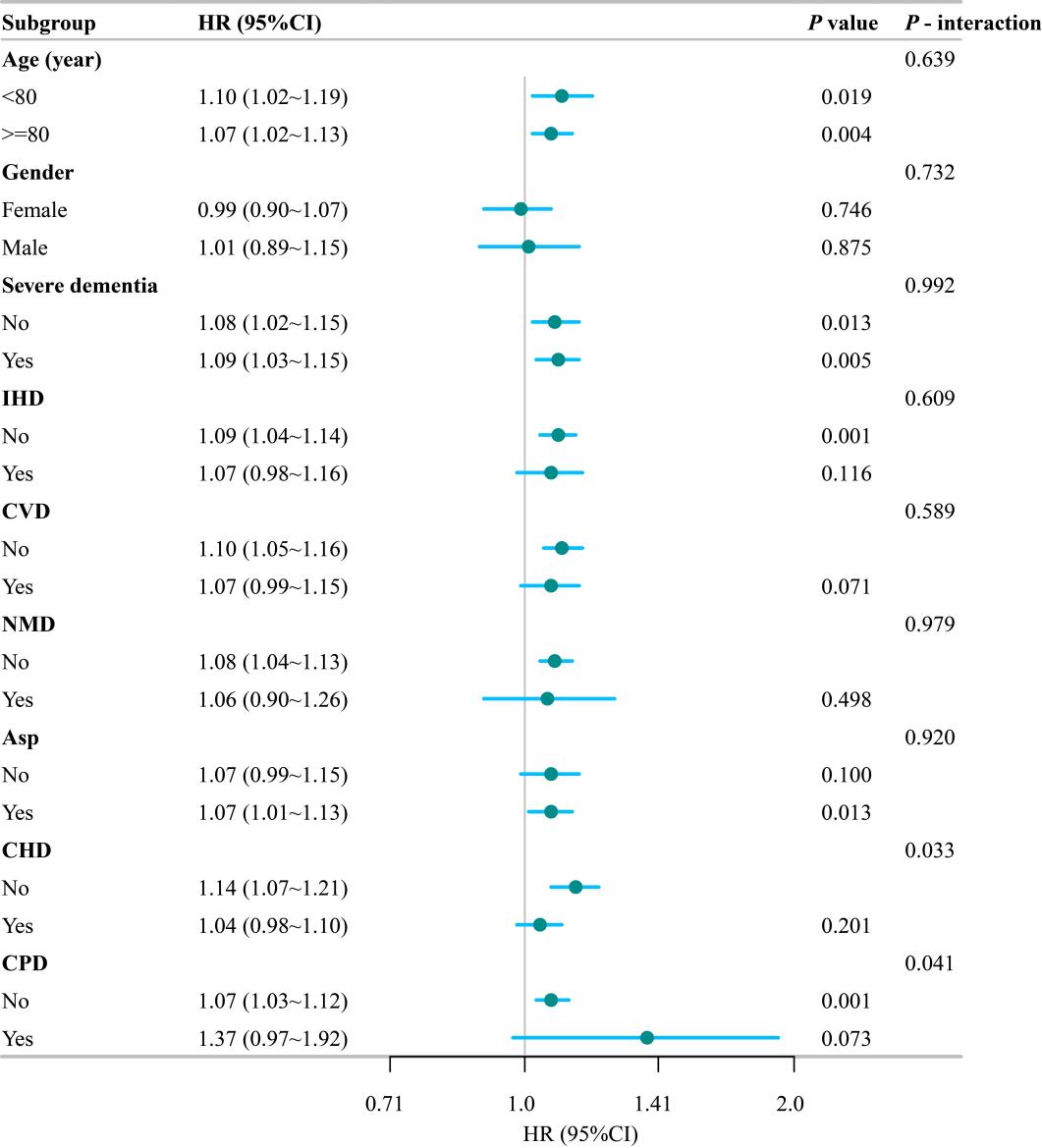


**Fig. S7** Subgroup analysis of the relationship between Deurenberg and pneumonia-cause.
